# Non‐Targeting shRNA‐Encoded Plasmid DNA Enhances Protective Immunity Through RIDD‐RIG‐I Signaling Pathway in the Zika Virus Animal Model

**DOI:** 10.1002/advs.202517420

**Published:** 2026-01-28

**Authors:** Min‐Syuan Huang, Hung‐Chun Liao, Po Peng, Wan‐Ling Wu, Kit Man Chai, Mei‐Yu Chen, Guann‐Yi Yu, Tsung‐Hsien Chuang, Hsin‐Wei Chen, Chuang‐Rung Chang, Shih‐Jen Liu

**Affiliations:** ^1^ National Institute of Infectious Disease and Vaccinology National Health Research Institutes Miaoli Taiwan; ^2^ Institute of Biotechnology College of Life Science and Medicine National Tsing Hua University Hsinchu Taiwan; ^3^ Welgene Biotech. Co., Ltd. Taipei Taiwan; ^4^ Immunology Research Center National Health Research Institutes Miaoli Taiwan; ^5^ Graduate Institute of Biomedical Sciences China Medical University Taichung Taiwan; ^6^ Graduate Institute of Medicine College of Medicine Kaohsiung Medical University Kaohsiung Taiwan

**Keywords:** immunogenicity, molecular adjuvants, non‐targeting shRNA, regulated IRE1‐dependent decay (RIDD) pathway, Zika virus

## Abstract

DNA vaccines offer advantages such as low production cost, rapid manufacturing, and enhanced stability for transport and storage, making them suitable for addressing tropical and emerging infectious diseases. However, their limited immunogenicity in humans often necessitates multiple booster doses or high antigen quantities, posing a significant barrier to broader application. To overcome this limitation, we developed a DNA vaccine encoding the Zika virus (ZIKV) structural proteins (prME) that incorporates a non‐targeting short hairpin RNA (shRNA) as an intrinsic molecular adjuvant. This shRNA is not designed to silence host genes; rather, it enhances innate immune “RIG‐I” signaling by selectively activating the IRE1α‐dependent regulated IRE1‐dependent decay pathway. Mechanistically, shRNA overexpression is inferred to increase the load on the RNA‐induced silencing complex, potentially triggering IRE1α oligomerization and subsequent upregulation of TXNIP. These events generate cytoplasmic RNA fragments that activate RIG‐I and downstream antiviral responses. In murine models, the vaccine elicited strong ZIKV‐specific humoral and cellular immune responses. In AGB6 mice, it significantly increased neutralizing antibody titers, reduced viremia, and improved survival. This approach enhances the immunogenicity of DNA vaccines without targeting host genes, providing a scalable and adaptable platform for infectious disease prevention.

## Graphical abstract

We present a novel DNA vaccine platform featuring intrinsic, non‐targeting dsRNA that significantly enhances immune responses by activating the regulated IRE1‐dependent decay‐RIG‐I signaling pathway. This research elucidates a previously uncharacterized mechanism for dsRNA‐mediated innate immune activation. By overcoming the historical limitations of low immunogenicity, this scalable and adaptable technology establishes a robust foundation for next‐generation DNA vaccines.

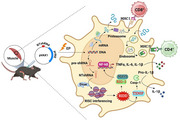

## 1 Introduction

DNA vaccines represent a promising nucleic acid‐based platform for combating infectious diseases and cancers, owing to their safety profile, stability at ambient temperature, cost‐effective production, and rapid manufacturing capacity [1, 2]. They elicit humoral and cellular immune responses by enabling the endogenous expression and presentation of antigens via MHC class I and II pathways [3]. Moreover, their versatility enables the incorporation of multiple antigens or immunomodulators into a single construct, thereby facilitating personalized and scalable vaccine development [4]. Nonetheless, the clinical success of DNA vaccines has been constrained by their suboptimal immunogenicity in humans, often necessitating multiple boosters, high doses, or potent adjuvants [5].

To overcome these limitations, previous studies explored co‐delivery of cytokines, chemokines, and pathogen‐associated molecular patterns (PAMPs) as adjuvants. While cytokines such as IL‐12 [6], IFN‐α [7], and IFN‐γ [6] can amplify immune activation, their overexpression may lead to adverse inflammatory responses [5, 7]. Similarly, PAMPs, such as CpG oligodeoxynucleotides [8], activate pattern recognition receptors, but often exhibit species‐specific effects, which complicates their translational application. These limitations underscore the need for novel molecular adjuvants that are both safe and effective and broadly applicable.

Beyond conventional adjuvants, we observed that non‐targeting siRNA induced immune responses in mouse bone marrow‐derived dendritic cells (mBMDCs), resulting in cytokine secretion and co‐stimulatory molecule expression. Additionally, we found that non‐targeting siRNA treatment activated the innate immune receptor RIG‐I. Previous research has also shown that non‐targeting siRNA upregulates RIG‐I in fibroblasts and enhances the clearance of doxorubicin‐induced senescent cancer cells [9]. In addition, an earlier study demonstrated that RIG‐I activation by short RNA ligands can protect human immune cells from Dengue virus infection without inducing cytotoxicity [10]. Like Dengue virus, another flavivirus, Zika virus, which spread and caused a pandemic in tropical and subtropical regions in 2015–2016, is a candidate for a prophylactic DNA vaccine [11].

We sought to determine how the non‐targeting siRNA induces immune responses in mouse mBMDCs and whether we could enhance the immunogenicity of our prophylactic Zika virus DNA vaccine by leveraging these responses.

To investigate how non‐targeting siRNA induces innate immune responses, we initially performed RNA sequencing in JAWSII cells, a mouse monocyte cell line, following siRNA transfection. This analysis revealed a marked upregulation of TXNIP (thioredoxin‐interacting protein), a stress‐responsive gene implicated in inflammatory signaling. Notably, gene set–level analyses further suggested coordinated transcriptional changes in pathways related to RNA silencing machinery and post‐transcriptional regulation, raising the possibility that non‐targeting siRNA accumulation perturbs RNA regulatory homeostasis rather than triggering a nonspecific stress response. Previous studies have linked TXNIP induction to activation of the IRE1α‐dependent regulated IRE1‐dependent decay (RIDD) pathway, in which selective RNA decay and microRNA turnover contribute to stress adaptation and innate immune signaling [12]. We examined whether this axis mediates the observed immune activation. Our findings suggest that disruption of RNA silencing dynamics by non‐targeting siRNA engages the IRE1α–RIDD pathway, leading to TXNIP upregulation and the generation of immunostimulatory RNA species that activate RIG‐I signaling. This novel link provided a mechanistic basis for incorporating non‐targeting siRNA as an intrinsic molecular adjuvant in a DNA vaccine platform.

Accordingly, we designed a DNA vaccine encoding the structural proteins of Zika virus (ZIKV), including the pre‐membrane (prM), membrane (M), and envelope (E) proteins, along with a non‐targeting short hairpin RNA (shRNA) to enhance immunogenicity. In murine models, this construct significantly boosted both antibody and T‐cell responses against ZIKV. Our study presents a novel approach to enhance DNA vaccine efficacy by leveraging the immunostimulatory potential of non‐targeting shRNA via RIDD‐mediated innate immune activation.

## 2 Results

### 2.1 The Non‐Targeting siRNA Transfection Activates Mouse DCs In Vitro

**FIGURE 1:**
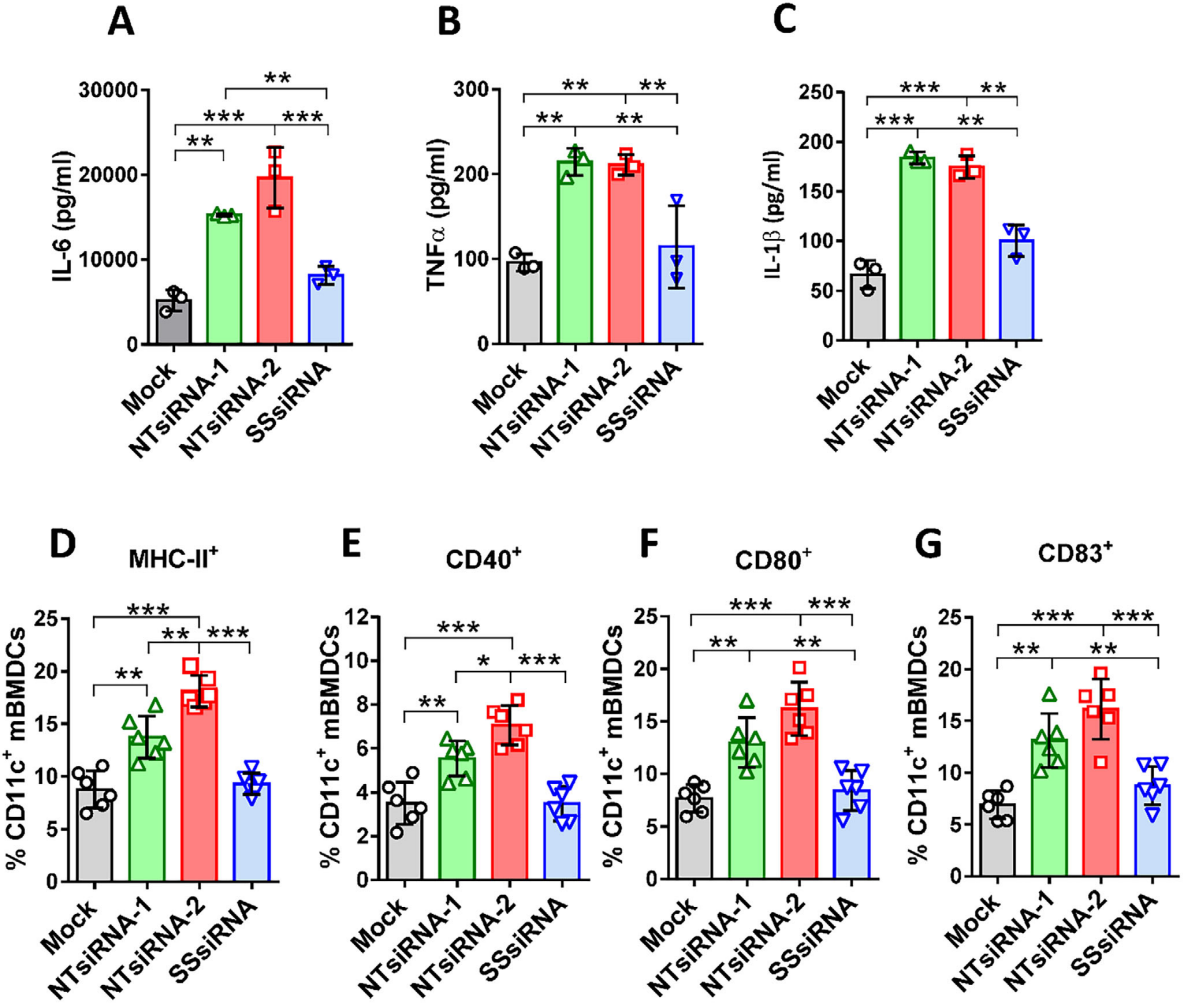
The non‐targeting siRNA expression activates dendritic cells. JAWSII cells were transfected with 300 pmol of NTsiRNA‐1, NTsiRNA‐2, or Single‐Stranded siRNA (SSsiRNA) for 48 h; the culture medium was then collected for analysis of cytokine secretion. The levels of (A) IL‐6, (B) TNFα, and (C) IL‐1β were determined by cytokine‐specific ELISA assay. Mouse bone marrow‐derived dendritic cells (BMDC) were transfected with 300 pmol of NTsiRNA‐1, NTsiRNA‐2, or SSsiRNA for 48 h, then immunostaining was performed to analyze the expression level of co‐stimulatory molecules (D) MHC‐II^+^, (E) CD40^+^, (F) CD80^+^, and (G) CD83^+^ on the cell surface by flow cytometry. Symbols represent data from each independent experiment. The data are shown as mean ± SD. Significance was calculated by one‐way ANOVA with Tukey's multiple comparisons. *P ≤ 0.05, **P ≤ 0.01, ***P ≤ 0.001, ****P ≤ 0.0001.

Nucleic acid sensors play a crucial role in mediating immune defense against pathogens and in detecting tumor‐derived DNA to initiate antitumor immune responses. In this study, we initially employed non‐targeting siRNAs (NTsi) to investigate the potential of short double‐stranded RNA as a molecular adjuvant to elicit immune responses by activating RNA nucleic acid sensors. To clarify whether non‐targeting siRNA accumulation could trigger immune responses in antigen‐presenting cells (APCs) such as dendritic cells (DCs) to secrete cytokine or activate co‐stimulatory signals, two non‐targeting siRNAs, NTsiRNA‐1 and NTsiRNA‐2, and the single‐strand siRNA (SSsiRNA) mixture of both NTsiRNA‐1 and NTsiRNA‐2 sequences were used to transfect into the JAWSII mouse DCs and mBMDC. As shown in (Figure 1A–C), the transfection of NTsiRNA‐1 and NTsiRNA‐2 induced JAWSII mouse DCs to secrete significantly higher levels of IL‐6, TNFα, and IL‐1β, as compared to the SSsiRNA and mock (transfection reagent TransIT‐X2 only). Additionally, the two non‐targeting siRNAs, NTsiRNA‐1 and NTsiRNA‐2, as well as SSsiRNA, were transfected into mBMDCs, and the cell‐surface co‐stimulatory molecules were analyzed by flow cytometry. As shown in (Figure 1D–G), the transfection of non‐targeting siRNAs activated mBMDC to increase MHC‐II, CD40, CD80, and CD83 expression on the cell surface. Since the expression of MHC‐II, CD40, and CD83 on mBMDC transfected with NTsiRNA‐2 is even statistically higher than NTsiRNA‐1, NTsiRNA‐2 could have a higher potential to activate DCs. On the other hand, transfection with SSsiRNA did not increase the expression of MHC‐II, CD40, CD80, and CD83 on the mBMDC cell surface compared with the mock control. Therefore, we employed NTsiRNA‐2 as the candidate molecular adjuvant for the following analysis. Moreover, the NTsiRNA‐2 sequence was used to design a non‐targeting shRNA‐encoded anti‐Zika virus DNA vaccine, pVax‐NTsh‐prME.

### 2.2 Non‐Targeting siRNA Treatment Induces Distinct Transcriptional Responses Associated With Immune and Stress Pathways

To characterize the global transcriptional effects of non‐targeting siRNA (NTsiRNA‐2) treatment, we performed RNA sequencing and conducted a comparative transcriptomic analysis with mock‐treated controls. A heat map of the top 50 differentially expressed genes (DEGs) revealed distinct clustering patterns between NTsiRNA‐2‐treated and control samples, indicating robust transcriptomic reprogramming (Figure S2). Upregulated genes included CD68, CD14, and CXCL2, associated with innate immune activation, and mitochondrial transcripts such as mt‐Co1, mt‐Nd1, and mt‐Nd2, suggesting enhanced oxidative activity. In contrast, several genes involved in ribosomal function and immune regulation were downregulated, including Rplp1, Rps15, Bst2, Wfdc21, B2m, and Chil3. These findings indicate that NTsiRNA‐2 treatment modulates innate immune homeostasis and cellular stress responses, affecting metabolic regulation and protein synthesis pathways.

**FIGURE 2:**
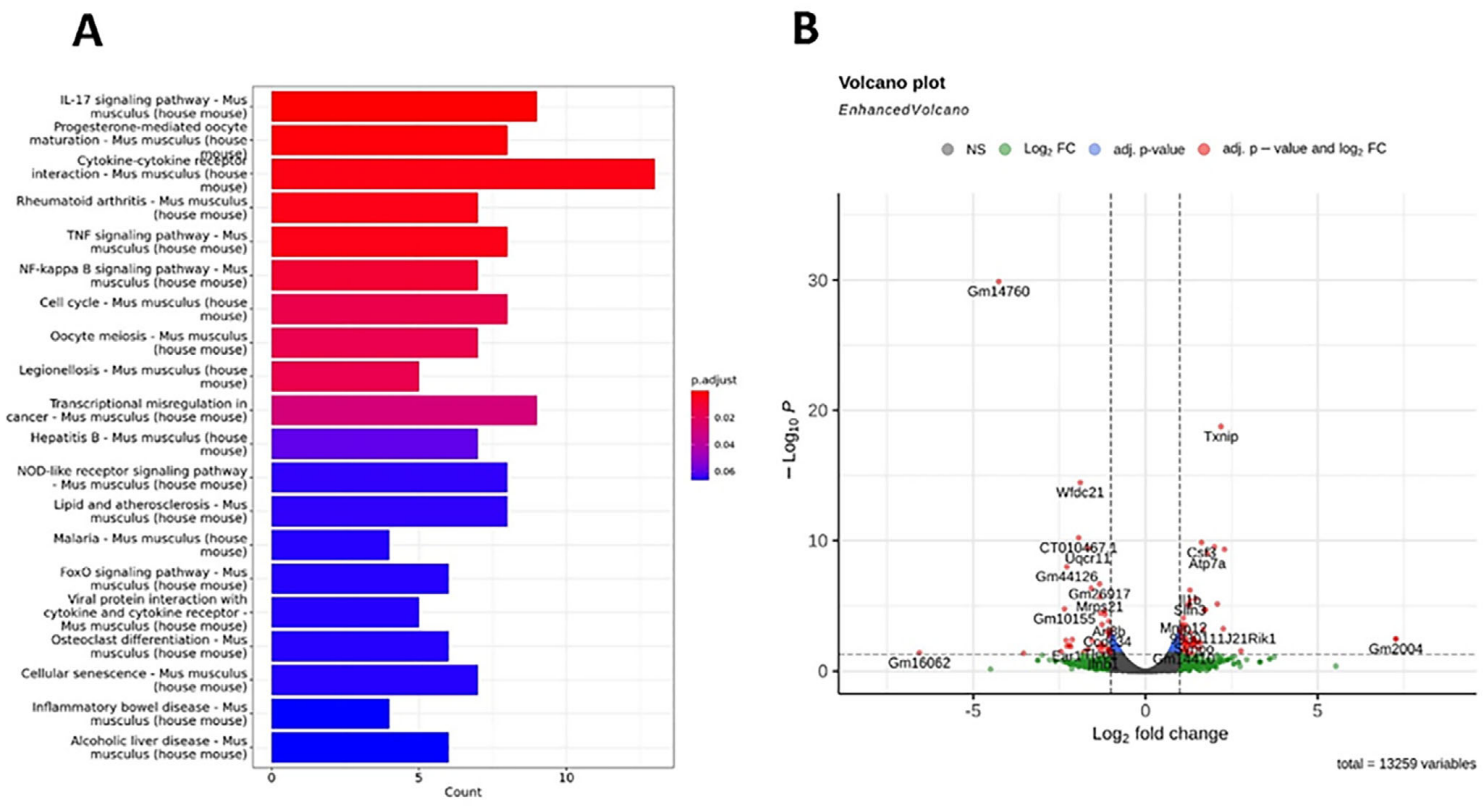
RNA‐sequencing analysis of JAWSII cells with or without non‐targeting siRNA expression. (A) Functional enrichment of differentially expressed genes (DEGs) with KEGG pathway analysis. RNA sequencing was performed with three biological replicates per group (*n* = 3). The significance of pathway enrichment was evaluated using adjusted *p*‐values (Benjamini–Hochberg correction), and results were visualized in a bar plot with color gradients indicating adjusted *p*‐values (red: low adjusted *p*‐value; blue: high adjusted *p*‐value). (B) A volcano plot of gene‐expression changes (fold change) and their statistical significance was generated by comparing gene‐expression variations between groups (*n* = 3 per group). The right side of the *x*‐axis represents genes with increased expression, while the left represents genes with decreased expression. The *y*‐axis shows genes with higher statistical significance toward the top. A total of 13 259 genes were analyzed. Genes with an adjusted *p*‐value < 0.05 and |log_2_ fold change| > 1 were considered significantly differentially expressed and are marked in red on the volcano plot.

Moreover, we performed KEGG pathway enrichment analysis using DEGs to gain insight into the biological pathways affected by NTsiRNA‐2 treatment. As shown in Figure 2A, a significant enrichment of immune‐ and inflammation‐associated pathways was observed. These included IL‐17 signaling, cytokine–cytokine receptor interaction, TNF signaling, NF‐κB signaling, and rheumatoid arthritis pathways, highlighting the activation of inflammatory networks typically involved in innate immune defense.

Further differential gene expression analysis was visualized using a volcano plot to assess statistical significance and fold changes (Figure 2B). Several genes were significantly upregulated, most notably thioredoxin‐interacting protein (TXNIP), a key mediator of oxidative and endoplasmic reticulum (ER) stress, as well as Csf3, Atp7a, Slfn3, Arl8b, and Wfdc21, which are associated with immune regulation and inflammation. The pronounced induction of TXNIP suggests it may serve as a central regulator of the transcriptional response elicited by non‐targeting siRNA transfection.

Collectively, these transcriptional changes indicate that non‐targeting siRNA treatment induces a coordinated immune‐ and stress‐associated gene expression program. However, whether this response reflects a generalized cellular stress reaction or a selective perturbation of RNA regulatory pathways remained unclear. To address this, we next performed gene set–level analyses to determine whether specific RNA silencing and ER stress–associated mechanisms are engaged by non‐targeting siRNAs.

### 2.3 GSEA Links Transcriptomic Signatures to RISC Perturbation and RIDD–RIG‐I Activation

**FIGURE 3:**
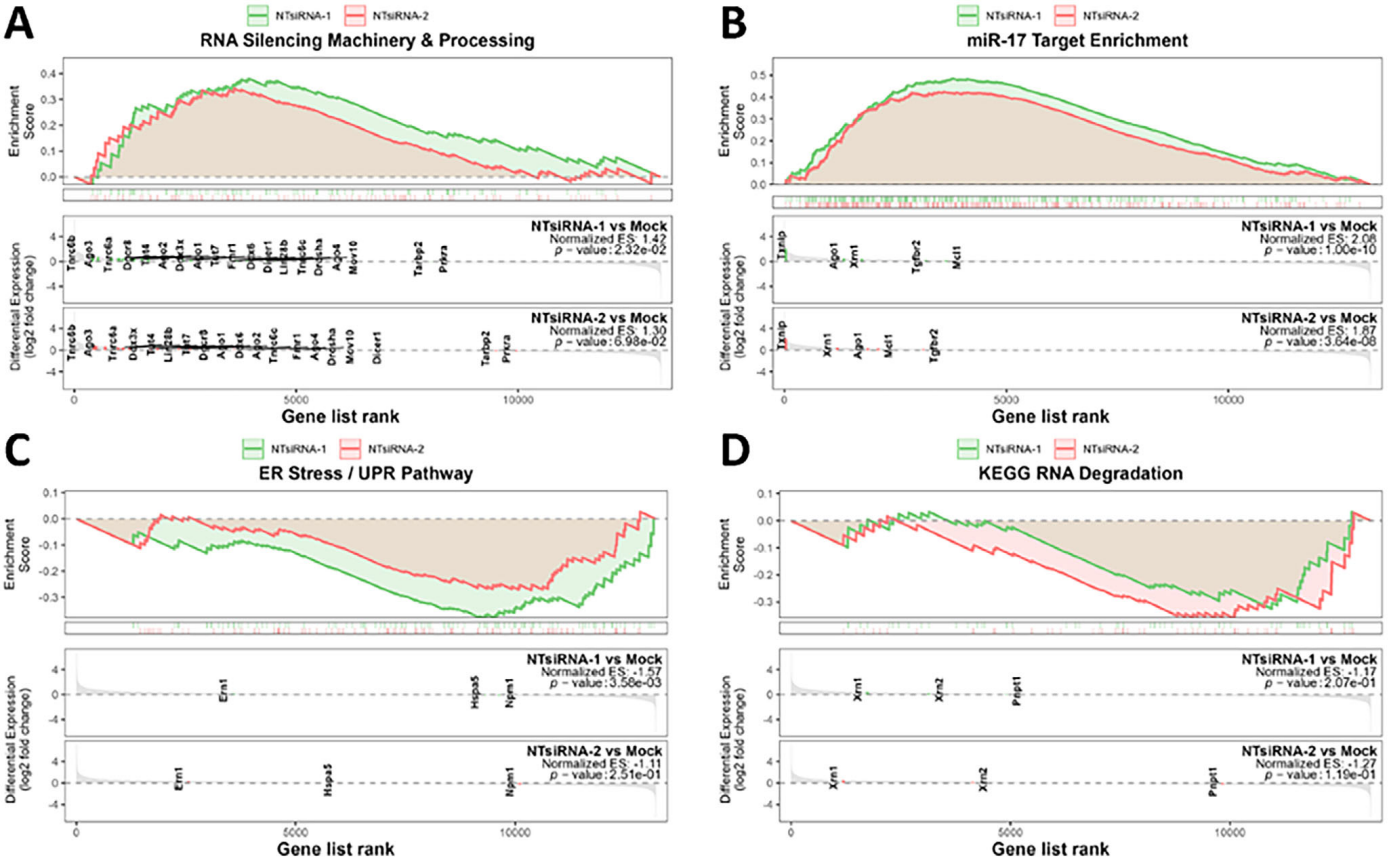
Gene set enrichment analysis (GSEA) reveals compensatory activation of RNA silencing machinery in non‐targeting siRNA‐transfected cells. GSEA plots showing the enrichment of the “RNA Silencing Machinery and Processing”, “miR‐17 target enrichment”, “Response to ER stress”, and “KEGG RNA degradation” gene sets. RNA sequencing was performed with three biological replicates per group (*n* = 3). The analysis compares transcriptomic profiles of JAWSII cells transfected with NTsiRNA‐1 (green) or NTsiRNA‐2 (red) against the Mock control. The top panel displays the enrichment score (ES), which indicates whether enrichment is positive or negative. The middle panel visualizes the distribution of gene set members (hits) within the ranked gene list. The bottom panels show the ranked log_2_ fold change (log_2_FC) of the leading‐edge genes for the NTsiRNA‐1 and NTsiRNA‐2 groups, respectively. Key genes, including Ago1, Txnip, Ern1, and Xrn2, are highlighted, demonstrating consistent transcriptional changes across both independent non‐targeting siRNA sequences. The normalized enrichment score (NES) and adjusted *p*‐values are indicated in the plots.

To further elucidate the mechanistic basis underlying the transcriptional reprogramming induced by non‐targeting siRNAs, we performed gene set enrichment analysis (GSEA) using RNA‐seq data from JAWSII cells transfected with NTsiRNA‐1 or NTsiRNA‐2 compared with mock controls (Figure 3). Notably, gene sets associated with RNA silencing machinery and processing were significantly enriched in both NTsiRNA‐treated groups, with coordinated upregulation of key RNA‐induced silencing complex (RISC)‐related components, including *Ago* and *Tnrc6* gene family, *Drosha*, and *Dicer* (Figure 3A). This enrichment was consistently observed across two independent non‐targeting siRNA sequences, suggesting a compensatory transcriptional response related to RNA silencing complex engagement rather than a sequence‐specific effect.

In parallel, GSEA revealed significant enrichment of miR‐17 target gene sets in both NTsiRNA‐1‐ and NTsiRNA‐2‐treated cells. Among the leading‐edge genes, *Txnip* [13] and *Tgfbr2* [14] (Figure 3B) were prominently upregulated, consistent with previous reports that IRE1α‐mediated regulated IRE1‐dependent decay (RIDD) selectively degrades miR‐17, thereby relieving post‐transcriptional repression of its target genes. Importantly, this transcriptional signature was not accompanied by enrichment of canonical unfolded protein response pathways, proteasome‐associated gene sets, or global RNA degradation programs, indicating that the observed response does not reflect non‐specific ER stress or widespread RNA decay.

Instead, we observed selective enrichment of non‐canonical ER stress–associated features, including increased expression of *Xrn2*, a 5′–3′ exoribonuclease previously linked to the resolution of cellular stress induced by non‐targeting siRNA (Figure 3D) [15]. Moreover, the gene encoding the ER stress sensor IRE1α (*Ern1*) was significantly upregulated in both NTsiRNA‐treated groups compared with mock controls (Figure 3C), positioning IRE1α as a central transcriptional node linking RNA silencing perturbation to downstream innate immune activation.

Collectively, these GSEA results support a model in which accumulation of non‐targeting siRNAs interferes with normal RISC dynamics, eliciting a compensatory transcriptional response in RNA silencing machinery and selectively engaging the IRE1α–RIDD axis. This transcriptional landscape provides a mechanistic framework linking the observed immune‐ and stress‐associated gene‐expression changes to the subsequent activation of RIG‐I signaling, as described below.

### 2.4 Non‐Targeting siRNAs Activate RIG‐I Signaling via IRE1α‐Dependent RIDD Pathway

**FIGURE 4:**
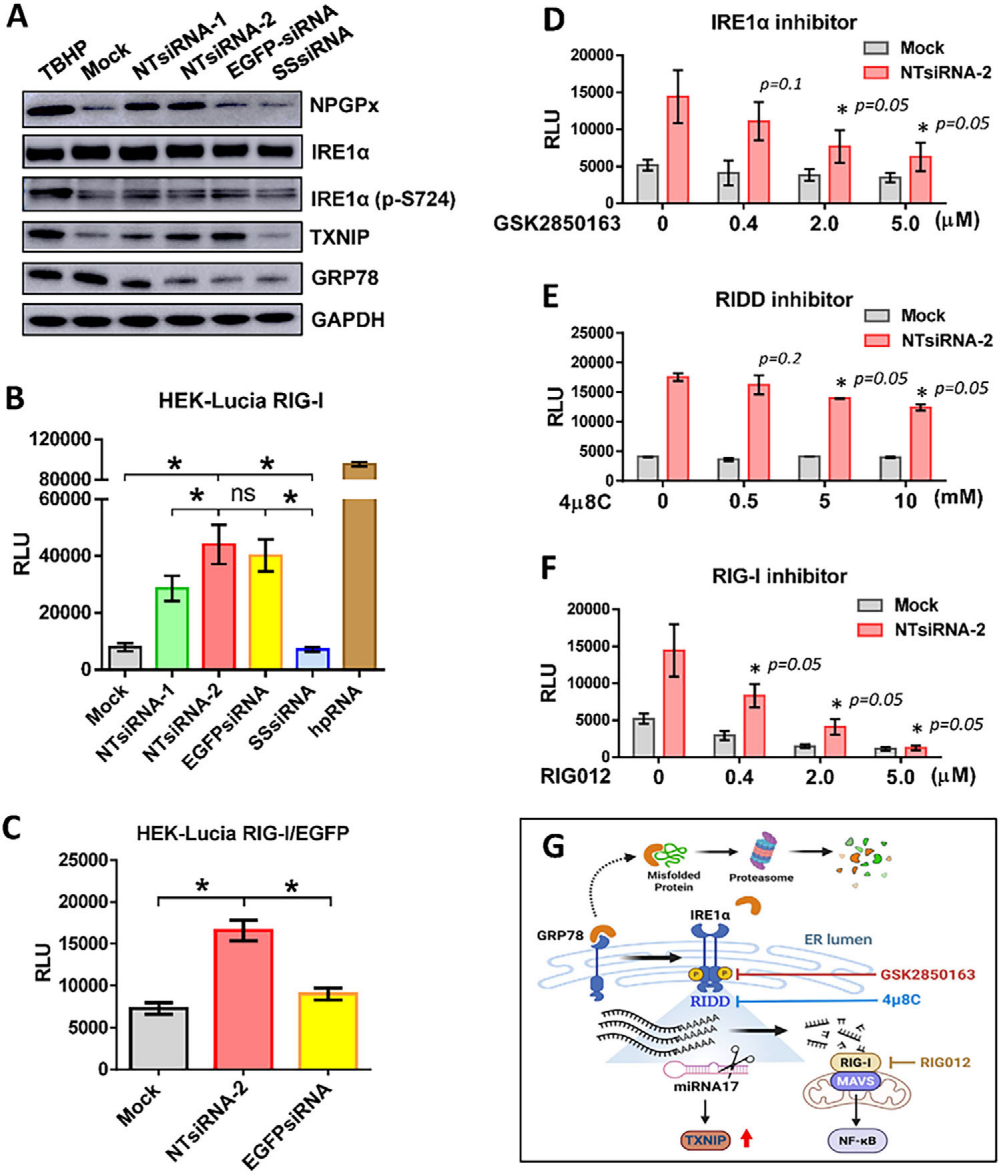
The non‐targeting siRNA expression activated the IRE1α‐RIG‐I signaling pathway. (A) HEK293 cells were transfected with NTsiRNAs and single‐stranded siRNA (SSsiRNA) for 24 h or incubated with TBHP (tert‐butyl hydroperoxide) for 6 h, and cell lysates were collected for western blotting analysis. The expression levels of NPGPx, IRE1α, IRE1α (p‐S724), TXNIP, GRP78, and GAPDH were detected using specific antibodies. (B–F) HEK‐Lucia RIG‐I or HEK‐Lucia RIG‐I/EGFP cells were transfected with NTsiRNAs, single‐stranded siRNA (SSsiRNA), EGFP siRNA, or positive controls (hpRNA). Where indicated, cells were treated with IRE1α inhibitor (0.4, 2, and 5 µm), RIG‐I inhibitor (0.4, 2, and 5 µm), or RIDD inhibitor (0.5, 5, and 10 mm) for 24 h following transfection. Supernatants were then collected for measurement of luciferase activity. All luciferase reporter assays were performed with three independent biological replicates per group (*n* = 3). Data are presented as mean ± SD from three independent experiments. Significance was calculated by Mann–Whitney two‐tailed *t*‐test comparisons. *p < 0.05, **p < 0.005, and ns = not significant. (G) Schematic model summarizing non‐targeting siRNA–induced activation of the IRE1α–RIDD–RIG‐I signaling axis. Excess non‐targeting siRNA interferes with RISC function, thereby activating and phosphorylating IRE1α, which triggers regulated IRE1‐dependent decay (RIDD). RIDD‐mediated degradation of miR‐17 relieves post‐transcriptional repression of TXNIP, while RNA decay products generated by RIDD activate RIG‐I and downstream MAVS‐dependent signaling. Pharmacological inhibitors targeting IRE1α kinase activity (GSK2850163), IRE1α RNase activity (4µ8C), or RIG‐I signaling (RIG012) attenuate this pathway, supporting the functional hierarchy of the IRE1α–RIDD–RIG‐I axis.

In our preliminary research, we inadvertently found that non‐targeting siRNA transfection induced RIG‐I activation in HEK‐Lucia RIG‐I cells (RIG‐I luciferase reporter cell line). The regulated IRE1α‐dependent decay (RIDD) pathway degrades miR‐17, thereby increasing thioredoxin‐interacting protein (TXNIP) expression. Additionally, RIDD activation generates small RNA fragments that activate retinoic acid‐inducible gene I (RIG‐I) and mitochondrial antiviral protein, thereby increasing IFN‐β production through the NF‐κB pathway [16]. Combined with the clue that the gene expression of TXNIP was significantly increased under NTsiRNA‐2 overexpression. We were curious to know whether non‐targeting siRNAs (NTsiRNAs) activate the RIG‐I signaling pathway through IRE1α‐dependent regulated IRE1‐dependent decay (RIDD). Phosphorylation of IRE1α at Ser724 induces its oligomerization and endoribonuclease activity, leading to degradation of selected mRNAs and microRNAs, including miR‐17, thereby upregulating TXNIP expression [17]. To further confirm that the double‐stranded siRNA is necessary for immune stimulation, we included a mixture of single‐stranded RNAs corresponding to the NTsiRNA‐1 and NTsiRNA‐2 sequences as a control. Western blot analysis confirmed our RNA‐seq findings, showing increased phosphorylated IRE1α (P‐S724) and TXNIP following transfection with NTsiRNA‐1, NTsiRNA‐2, and EGFPsiRNA. As a positive control, TBHP, a known ER stress inducer, also elevated IRE1α phosphorylation and TXNIP expression (Figure 4A). On the other hand, NPGPx, an ER‐resident stress‐responsive protein, was significantly induced following overexpression of non‐targeting siRNAs. However, GRP78 expression, a key chaperone and stress‐response protein, decreased after transfection with NTsiRNAs. We observed that single‐stranded siRNA (SSsiRNA) did not follow the same trend as NTsiRNA‐1 or NTsiRNA‐2. Conversely, SSsiRNA did not enhance IRE1α phosphorylation (P‐S724) or TXNIP or NPGPx expression (Figure 4A). These results suggest that NTsiRNAs selectively activate the IRE1α–RIDD axis without fully engaging the canonical unfolded protein response, thereby accumulating cytoplasmic RNA fragments lacking self‐associated markers. This process subsequently activates RIG‐I and downstream NF‐κB and type I interferon signaling, promoting a pro‐inflammatory antiviral state [17].

To assess whether non‐targeting siRNA could activate RIG‐I signaling via the IRE1α–RIDD axis, HEK‐Lucia RIG‐I cells (RIG‐I luciferase reporter cell line) were transfected with various non‐targeting siRNAs, such as NTsiRNA‐1, NTsiRNA‐2, and EGFPsiRNA. Single‐stranded siRNA (SSsiRNA) was also included as a control to evaluate the RIG‐I luciferase reporter assay. NTsiRNA‐2 significantly increased luciferase secretion compared to mock, SSsiRNA, and NTsiRNA‐1. Similarly, EGFPsiRNA shows a pattern similar to that of EGFPsiRNA, significantly increasing luciferase secretion compared with mock, NTsiRNA‐1, and SSsiRNA (Figure 4B).

To confirm that the activation of RIG‐I signaling is indeed induced by the NTsiRNAs, we transfected EGFPsiRNA with HEK‐Lucia RIG‐I/EGFP cells, the EGFP stably expressed HEK‐Lucia RIG‐I cells, and the luciferase activity of the EGFPsiRNA‐transfected group was significantly reduced to around 50% as compared to the NTsiRNA‐2‐transfected group (Figure 4C). The GFP fluorescence expression ratio of HEK‐Lucia RIG‐I/EGFP cells was determined by flow cytometry; after being transfected with EGFPsiRNA, the population of EGFP‐expressed HEK‐Lucia RIG‐I/EGFP cells was reduced (Figure S3).

To define the signaling hierarchy underlying non‐targeting siRNA–induced RIG‐I activation, HEK‐Lucia RIG‐I cells were treated with pharmacological inhibitors targeting sequential components of the proposed pathway. NTsiRNA‐2 robustly increased RIG‐I–dependent luciferase activity compared with mock controls. This induction was significantly attenuated in a dose‐dependent manner by the IRE1α kinase inhibitor GSK2850163 (Figure 4D), indicating that IRE1α activation is required for NTsiRNA‐induced RIG‐I signaling.

To further determine whether the RNase activity of IRE1α is involved, cells were treated with the selective IRE1α RNase/RIDD inhibitor 4µ8C. Inhibition of RIDD activity similarly suppressed NTsiRNA‐2–induced luciferase activation in a dose‐dependent manner (Figure 4E), demonstrating that IRE1α‐mediated RNA decay is a critical downstream step in this pathway.

Finally, to confirm that the observed signaling converges on RIG‐I, cells were co‐treated with the RIG‐I inhibitor RIG012. Pharmacological blockade of RIG‐I significantly reduced NTsiRNA‐2–induced luciferase activity (Figure 4F), verifying that RIG‐I functions as the downstream effector of the IRE1α–RIDD axis.

Collectively, these results establish a sequential signaling cascade in which non‐targeting siRNA activates IRE1α, engages RIDD‐dependent RNA processing, and culminates in RIG‐I–mediated innate immune signaling, as summarized schematically in Figure 4G.

### 2.5 Non‐Targeting shRNA‐Encoding DNA Constructs Activate RIG‐I Signaling and are Compatible With ZIKV Antigen Expression

To evaluate the potential of non‐targeting shRNA (NTshRNA) as an intrinsic molecular adjuvant in DNA vaccines, we ligated the NTshRNA‐expressing DNA sequence downstream of the U6 promoter, which drives explicit shRNA expression. PCR‐amplified U6‐NTshRNA fragments were transfected into HEK‐Lucia RIG‐I cells. Compared with mock and U6‐only controls, U6‐NTshRNA transfection significantly increased RIG‐I‐dependent luciferase activity (Figure S4A) but not TLR3 signaling (Figure S4B). The U6‐NTshRNA fragment was cloned into a pVAX1‐based eGFP‐expressing plasmid (pVAX‐NTsh‐eGFP) to mimic a DNA vaccine configuration. Transfection with the complete U6‐NTshRNA plasmid also induced robust RIG‐I activation (Figure S4C) without TLR3 activation (Figure S4D), confirming that U6‐NTshRNA DNA can stimulate innate immune signaling via RIG‐I, similar to NTsiRNA.

To validate this adjuvant effect in a vaccine context, we constructed DNA vaccines encoding ZIKV prME structural proteins, with or without co‐expressed NTshRNA. The prME gene from the Puerto Rican ZIKV strain (PRVABC59) was codon‐optimized, and its native signal sequence was replaced with that of the Japanese Encephalitis Virus (Figure S5A,B). The constructs pVax‐prME and pVax‐NTsh‐prME were verified by restriction digestion (Figure S5C,D), and prME protein expression was confirmed by immunoblotting in HEK293T cells (Figure S5E), demonstrating successful antigen production from both constructs.

### 2.6 Non‐Targeting shRNA‐Encoded DNA Vaccine Elicits Stronger Humoral and Cellular Immune Responses in Mice

**FIGURE 5:**
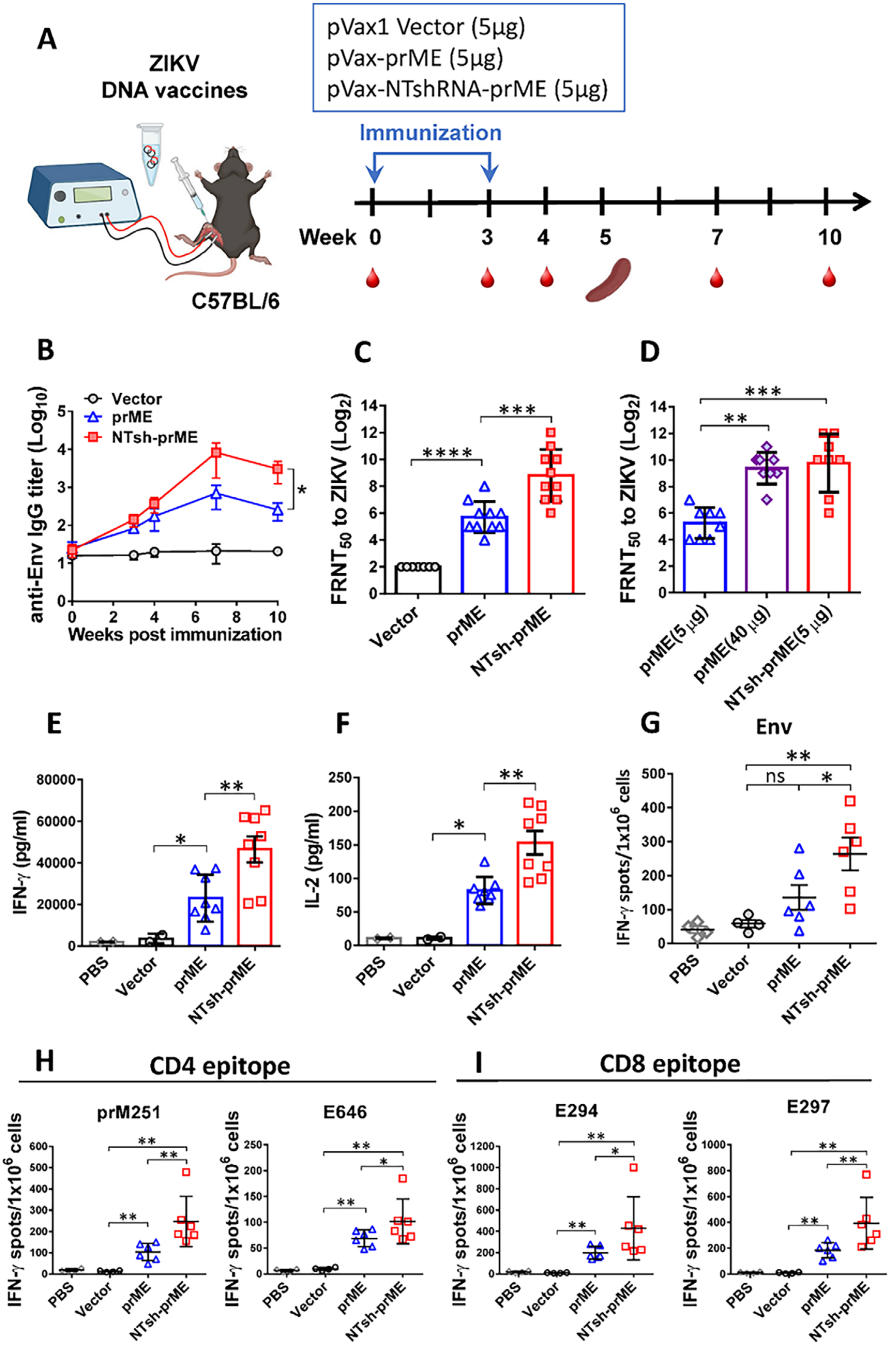
ZIKV DNA vaccine encoding non‐targeting shRNA enhances antibody and T‐cell responses. (A) C57BL/6 mice (*N* = 6–10 per group) were immunized with 5 µg DNA of pVax1 vector, pVax‐prME, pVax‐NTsh‐prME, and 40 µg DNA of pVax‐prME by the i.m. route with electroporation at weeks 0 and 3. Serums were collected at the indicated time points up to 10 weeks after the first immunization. Splenocytes were isolated two weeks after the second immunization to analyze antigen‐specific T‐cell immune responses. (B) The kinetics of the anti‐Env IgG titer were evaluated by using indirect ELISA. (C,D) The production of ZIKV‐neutralizing antibodies in serum collected at week 7 was assessed by measuring FRNT50. The levels of Th1 cytokines (E) IFN‐γ and (F) IL‐2 were determined by cytokine‐specific ELISA assays after Env stimulation for 48 h. In response to the stimulation of (G) Env and its (H) CD4 or (I) CD8 epitopes, the frequency of Env‐specific IFN‐γ‐secreting T cells (spot‐forming units per million splenocytes) induced in vaccinated mice was determined by IFN‐γ ELISpot assay. Each point represents individual animals, each bar reflects the mean, and error bars represent the standard deviation. Significance was calculated by Mann–Whitney one‐tailed *t‐*test comparisons. * *p* < 0.05, **P ≤ 0.01, ***P ≤ 0.001, ****P ≤ 0.0001.

To evaluate the adjuvant potential of intrinsic non‐targeting shRNA in enhancing DNA vaccine immunogenicity in vivo, C57BL/6 mice (*n* = 8/group) were immunized intramuscularly with electroporation at weeks 0 and 3 using 5 µg of pVax1, pVax‐prME, pVax‐NTsh‐prME, or 40 µg of pVax‐prME (Figure 5A). Humoral responses were assessed by measuring anti‐ZIKV envelope (Env)‐specific IgG titers in serum via ELISA. Antibody levels increased over time and peaked at week 7 post‐prime‐boost. Mice receiving pVax‐NTsh‐prME generated significantly higher anti‐Env IgG titers than the pVax‐prME group at weeks 7 and 10 (Figure 5B) and showed enhanced ZIKV neutralization titers (Figure 5C). Notably, 5 µg of pVax‐NTsh‐prME elicited neutralization titers comparable to 40 µg of pVax‐prME (Figure 5D).

Cell‐mediated immune responses were evaluated by cytokine secretion and T‐cell activation. Splenocytes from pVax‐NTsh‐prME‐immunized mice produced significantly higher levels of IFN‐γ (Figure 5E) and IL‐2 (Figure 5F) following Env protein re‐stimulation. ELISpot assays further revealed that pVax‐NTsh‐prME induced higher frequencies of IFN‐γ‐secreting cells than both the pVax‐prME and vector control groups after restimulation with purified Env, CD4 (prM251, E646), and CD8 (E294, E297) epitopes (Figure 5G–I). These results demonstrate that co‐expression of non‐targeting shRNA enhances both humoral and T‐cell‐mediated responses, supporting its role as a molecular adjuvant in DNA vaccination.

### 2.7 Non‐Targeting shRNA Enhances the Immunogenicity of a ZIKV DNA Vaccine in TLR3‐Knockout Mice but not in MAVS‐ or Caspase‐1‐Knockout Mice

To determine whether the immunostimulatory effect of NTshRNA‐encoded DNA vaccine is mediated via the IRE1α–RIDD–RIG‐I signaling axis, pVax‐NTsh‐prME and pVax‐prME constructs were administered intramuscularly into immunodeficient mice lacking key innate immune components, including TLR3^−^/^−^, MAVS^−^/^−^, and Casp1^−^/^−^ strains.

**FIGURE 6:**
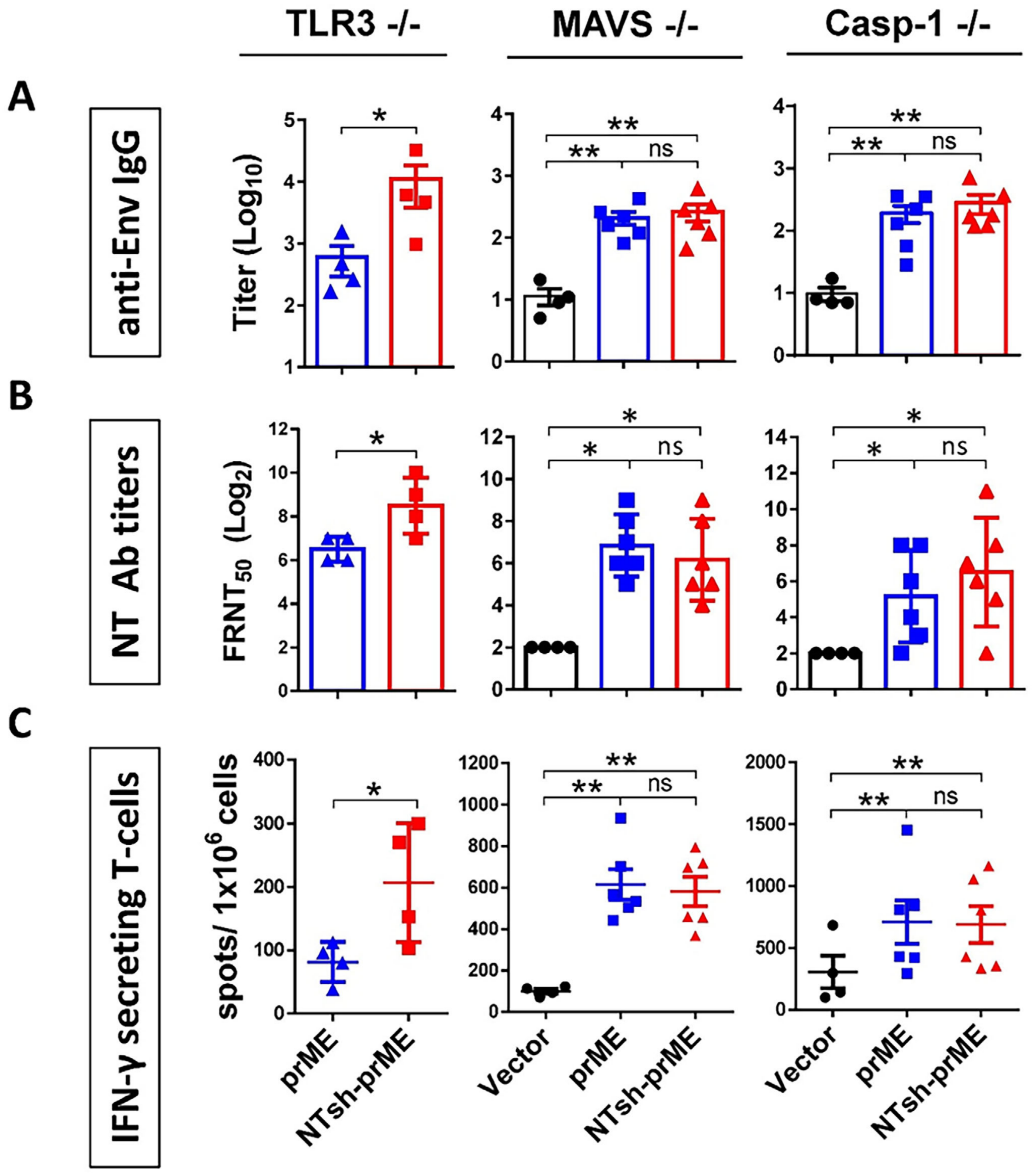
Non‐targeting shRNA enhances the immunogenicity of ZIKV DNA vaccine in TLR3 KO mice but not in MAVS and Caspase‐1 knockout mice. TLR3 KO, MAVS KO, and Caspase‐1 KO mice (*N* = 4–6 per group) were i.m. immunized with 5 µg DNA of pVax‐prME, or pVax‐NTsh‐prME by electroporation at weeks 0 and 3. Serum samples were collected at week 5 following the first immunization. (A) Anti‐Env IgG titers were measured by indirect ELISA. (B) The level of ZIKV‐neutralizing antibodies was quantified using the FRNT50 assay. Splenocytes were isolated to analyze antigen‐specific T‐cell immune responses. After the stimulation of Env for 48 h, (C) the frequency of Env‐specific IFN‐γ‐secreting T cells (spot‐forming units per million splenocytes) induced in vaccinated mice was determined by IFN‐γ ELISpot assay. Each point represents individual animals, each bar reflects the mean of titers, and error bars represent the standard deviation. Significance was calculated by Mann–Whitney one‐tailed *t*‐test comparisons. * *p* < 0.05, **P ≤ 0.01, and ns = no significant.

At week 5 post‐prime‐boost immunization, ZIKV‐specific anti‐Env IgG titers were measured by endpoint ELISA. In TLR3^−^/^−^ mice, pVax‐NTsh‐prME elicited significantly higher IgG titers than pVax‐prME, while no significant differences were observed in MAVS^−^/^−^ or Casp1^−^/^−^ mice (Figure 6A). Similarly, neutralizing antibody titers were significantly elevated in TLR3^−^/^−^ mice receiving pVax‐NTsh‐prME, but not in MAVS^−^/^−^ or Casp1^−^/^−^ groups (Figure 6B), indicating that MAVS and Caspase‐1 are required for the NTshRNA‐induced humoral response.

Cellular immunity was evaluated by IFN‐γ ELISpot assay following in vitro restimulation with purified Env protein (Figure 6C) or with specific T‐cell epitopes (Figure S6). Only TLR3^−^/^−^ mice vaccinated with pVax‐NTsh‐prME exhibited a significant increase in IFN‐γ‐secreting cells (Figure 6C). In contrast, no significant differences were observed in MAVS^−^/^−^ or Casp1^−^/^−^ mice (Figure 6C). These findings indicate that the enhanced humoral and cellular immune responses induced by NTshRNA depend on MAVS and Caspase‐1 signaling but are not significantly affected by TLR3 deficiency.

### 2.8 NTshRNA‐Encoding DNA Vaccine Confers Dose‐Dependent Protection Against ZIKV Challenge in AGB6 Mice

**FIGURE 7:**
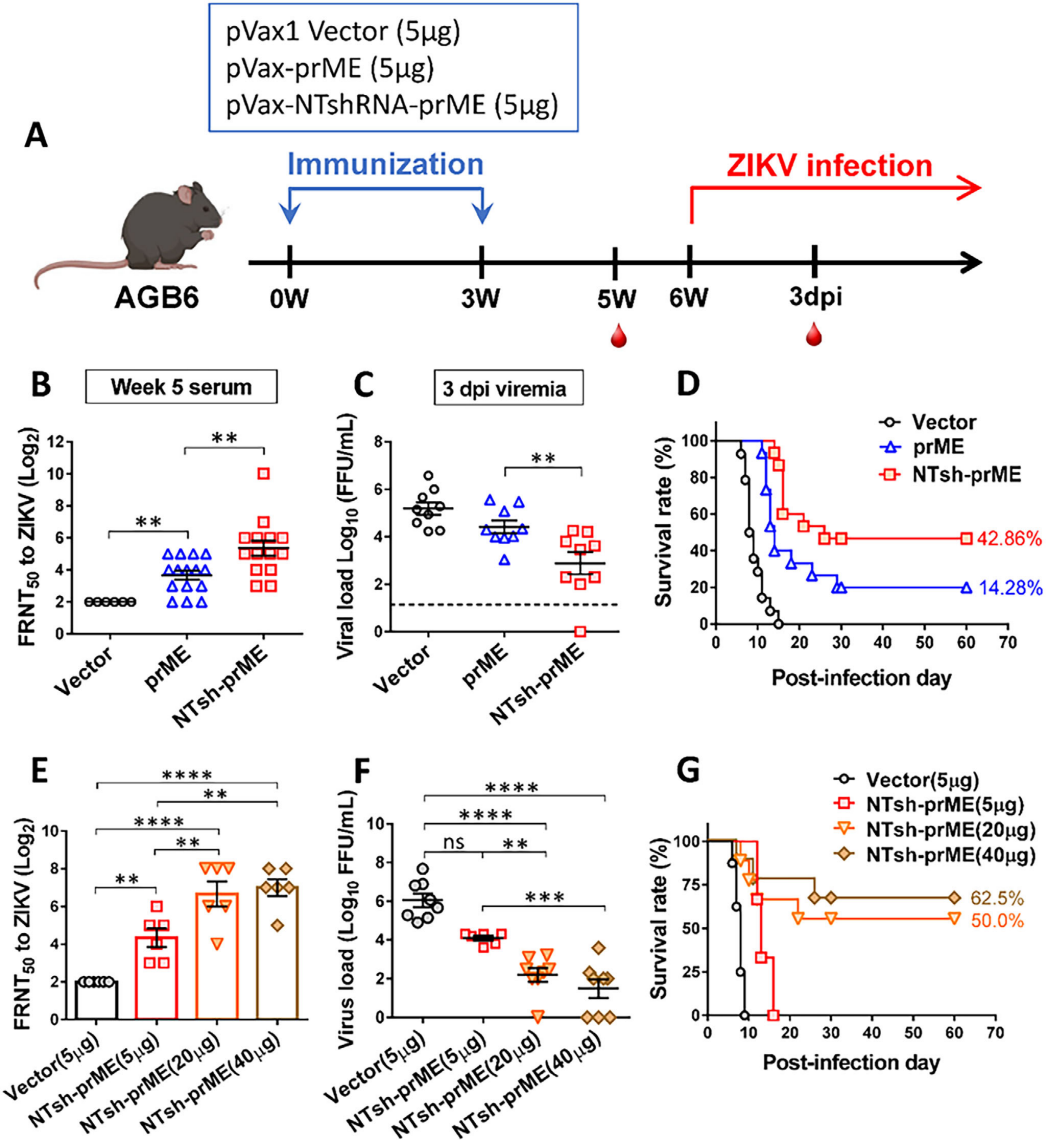
Immunization of NTshRNA‐encoded ZIKV DNA vaccine protects AGB6 mice against ZIKV infection. (A) Schematic schedule of animal study in AGB6 mice. AGB6 mice were immunized i.m. twice with 5 µg of empty vector (pVax1), pVax‐prME, and pVax‐NTsh‐prME by electroporation at weeks 0 and 3, and then i.p. challenged with ZIKV at week 6. (B,E) ZIKV‐neutralizing antibody titers were evaluated by calculating the FRNT50 at week 5 after the first immunization. AGB6 mice were i.p. challenged with (C,D) 50 FFU ZIKV or (F,G) 100 FFU ZIKV at week 6. (C,F) The viral load on 3 days post‐infection (dpi) was determined by calculating the FRNT50. Each point represents an individual animal, and the horizontal bar indicates the mean and standard deviation for each group. (D,G) Survival rates for each group were recorded continuously after the ZIKV challenge until 30 dpi. Significance was calculated by one‐way ANOVA with Tukey's multiple comparisons. **P ≤ 0.01, ***P ≤ 0.001, ****P ≤ 0.0001, and n.s = no significant.

As C57BL/6 mice are resistant to symptomatic ZIKV infection, AGB6 mice‐C57BL/6‐derived transgenic mice lacking type I and II interferon receptors—were used to evaluate the protective efficacy of the DNA vaccine. AGB6 mice were immunized intramuscularly with 5 µg of pVax1, pVax‐prME, or pVax‐NTsh‐prME, following the same regimen as in C57BL/6 mice, and challenged intraperitoneally with 50 FFU ZIKV six weeks after priming. Serum samples were collected one week before ZIKV challenge and three days post‐challenge to assess neutralizing antibody titers and quantify viremia (Figure 7A).

Compared to pVax‐prME, pVax‐NTsh‐prME induced significantly higher neutralizing antibody titers (Figure 7B) and reduced viremia (Log 2.88 vs. Log 4.42 FFU/mL; Figure 7C). Survival analysis showed that all vector control mice succumbed by 15 dpi, and only 2 of 14 mice immunized with pVax‐prME survived by 30 dpi (14.28%). In contrast, pVax‐NTsh‐prME improved survival to 42.86% (6 of 14 mice; Figure 7D).

To evaluate the protective efficacy and safety of the pVax‐NTsh‐prME DNA vaccine, AGB6 mice were immunized with 5, 20, or 40 µg of the vaccine and subsequently challenged with a higher ZIKV dose of 100 FFU, twice the viral load used in prior experiments, to assess dose‐dependent protection under more stringent conditions. Neutralizing antibody titers increased with dose (Figure 7E), with 20 µg inducing an 8‐fold increase relative to 5 µg and reducing viral load to approximately Log 2 FFU/mL (Figure 7F). While neutralization titers and viral load showed no significant differences between 20 and 40 µg, the 40 µg group had a ∼20% higher survival rate (Figure 7G). These results demonstrate dose‐dependent immune protection by NTshRNA‐encoded DNA vaccine without observable toxicity.

**FIGURE 8:**
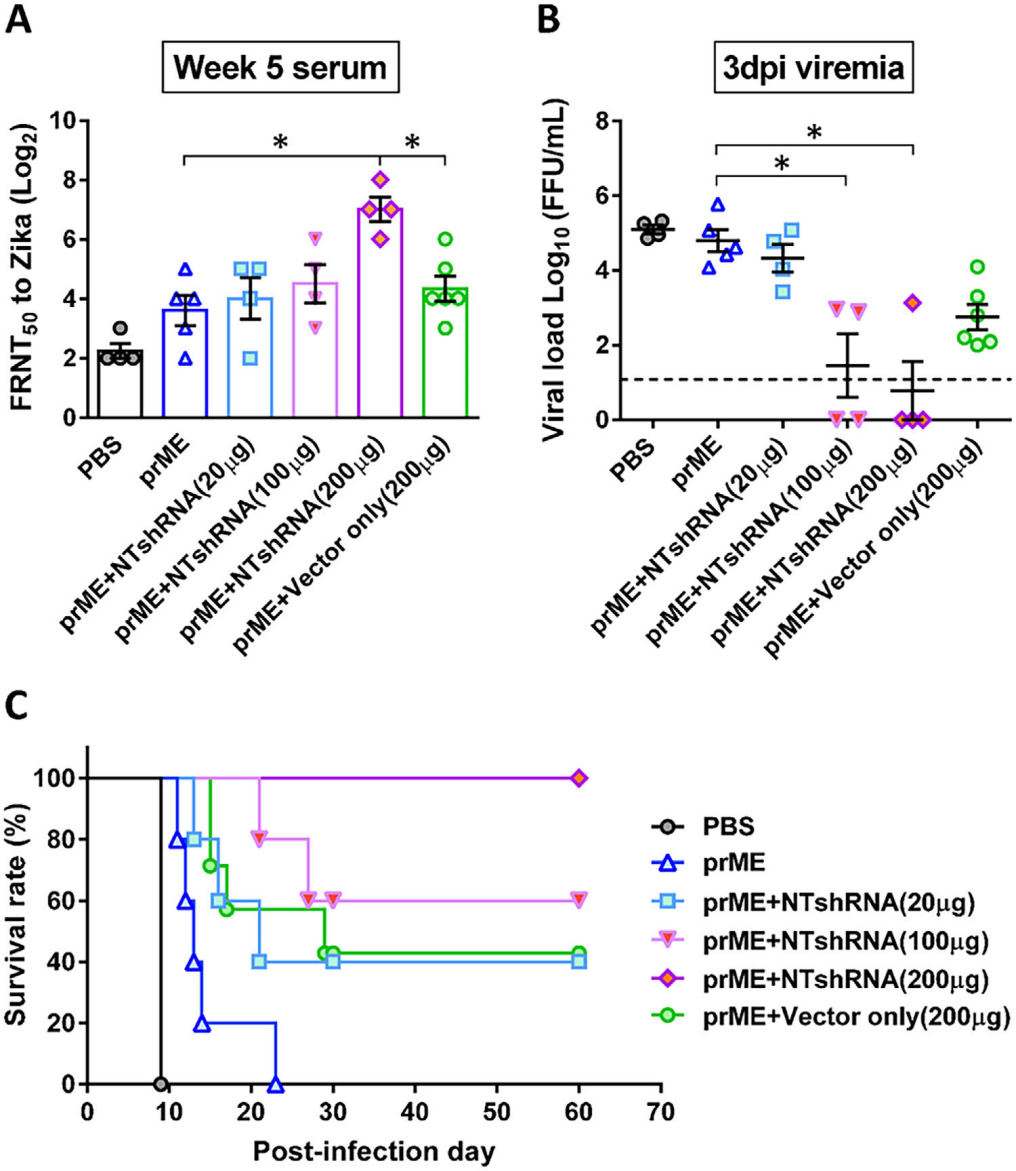
The adjuvant effect of the non‐targeting shRNA encoded ZIKV DNA vaccine was indeed attributed to the expression of the non‐targeting shRNA, as the protective efficacy of the ZIKV DNA vaccine was improved by co‐delivery of the NTshRNA‐expressing vector in a dose‐dependent manner. AGB6 mice were i.m. immunized twice with 5 µg of pVax‐prME with or without NT shRNA‐expressing vector (pLKO‐NTshRNA) with doses of 20, 100, and 200 µg by electroporation at week 0 and 3, then i.p. challenged with ZIKV at week 6. (A) Serum was collected 2 weeks after the second immunization and used to assess neutralizing activities by counting FRNT50. AGB6 mice were i.p. challenged with 50 FFU ZIKV at week 6. (B) Serum was collected and diluted 3 days post‐infection (dpi) to determine viral load by measuring FRNT50. (C) Mouse survival rate was tracked for 30 days post‐ZIKV infection. * *p* < 0.05, ** *p* < 0.005, *** *p *< 0.0005, **** *p* < 0.0001.

To further verify whether the observed immune protection was directly linked to NTshRNA expression, due to the concern that it's difficult to quantify how effective the transcript was, as the non‐targeting shRNA expression is difficult to detect in vivo. A question may arise as to whether the observed protective responses were induced by CpG effects arising from the vector backbone. AGB6 mice were co‐immunized with a fixed dose of prME‐encoding plasmid and increasing doses of non‐targeting shRNA‐expressing plasmid (pLKO‐TRC). Neutralizing antibody titers increased (Figure 8A), and viral load decreased (Figure 8B) in a dose‐dependent manner. Notably, higher NTshRNA doses conferred complete (100%) protection even at a low antigen dose of 5 µg (Figure 8C). These findings confirm that the intrinsic adjuvant effect is mediated by the in vivo expression level of the non‐targeting shRNA.

## 3 Discussion

This study demonstrates that non‐targeting shRNA enhances DNA vaccine immunogenicity by indirectly activating RIG‐I via the IRE1α‐dependent regulated IRE1‐dependent decay (RIDD) pathway. We propose that excessive expression of non‐targeting siRNA perturbs RNA silencing homeostasis by overloading the RNA‐induced silencing complex (RISC), thereby triggering a stress‐adaptive response mediated by the ER stress sensor IRE1α. Consistent with this model, GSEA revealed a coordinated transcriptional upregulation of multiple core components of the RNA silencing machinery, including members of the Argonaute (Ago) and Tnrc6 families, as well as Drosha and Dicer, in cells transfected with two independent non‐targeting siRNA sequences compared with mock controls (Figure 3). This enrichment pattern suggests a compensatory response to increased RNA silencing demand rather than sequence‐specific off‐target effects.

Importantly, the GSEA signatures did not show enrichment of canonical unfolded protein response (UPR), proteasome, or global RNA degradation pathways, arguing against nonspecific ER stress or widespread RNA decay. Instead, selective enrichment of non‐canonical stress‐associated features, including the exoribonuclease Xrn2 [15] and the IRE1α‐encoding gene Ern1, was observed in both non‐targeting siRNA groups. Together, these transcriptomic features support a model in which disruption of RISC dynamics selectively engages the IRE1α–RIDD axis rather than eliciting a generalized stress response.

Consistent with this interpretation, luciferase activity induced by non‐targeting siRNAs was significantly attenuated in HEK‐Lucia RIG‐I/EGFP cells transfected with EGFP siRNA, in which the presence of a cognate target alleviates RISC saturation and reduces stress‐associated innate signaling. Additionally, Western blot analysis confirmed the upregulation of NPGPx—an ER‐resident protein known to resolve siRNA‐induced stress—following non‐targeting siRNA transfection. NPGPx is induced selectively by non‐targeting siRNAs but not by canonical ER stressors and functions independently of GRP78, calnexin, or XBP1 activation [15]. Consistent with a RIDD‐driven process, GRP78 protein levels were reduced following non‐targeting siRNA treatment, potentially due to selective mRNA cleavage, as GRP78 transcripts harbor IRE1α‐sensitive cleavage motifs [18]. Collectively, these data suggest that NPGPx may act upstream of IRE1α as a buffering regulator during RNA overload, facilitating engagement of a distinct ER stress signature that culminates in RIDD activation and downstream RIG‐I signaling [19].

To exclude the possibility that the observed immunostimulatory effects were driven by intrinsic siRNA sequence or structural features, we performed additional validation analyses. Motif mapping and secondary‐structure predictions indicated that the non‐targeting siRNAs used in this study did not exhibit enrichment for known immunostimulatory elements, such as CpG motifs, GU‐rich sequences, or atypical structural configurations associated with innate immune activation (Figure S7). Moreover, inclusion of an additional unrelated non‐targeting LacZ siRNA recapitulated the activation of the IRE1α–RIDD–RIG‐I axis, confirming that this phenomenon is broadly associated with non‐targeting siRNA expression rather than being restricted to a specific sequence or structural motif (Figure S8). These findings further support a sequence‐independent mechanism driven by perturbation of RNA silencing homeostasis.

RIG‐I, a key pattern recognition receptor, orchestrates antiviral innate immunity and protects immune cells against RNA viruses such as Dengue [10]. In this study, co‐expression of non‐targeting shRNA with ZIKV prM/E antigens significantly enhanced humoral and cellular immune responses, including elevated ZIKV‐specific antibody titers, increased neutralization activity, augmented Th1 cytokine production, and higher frequencies of IFN–γ–secreting T cells. The immunostimulatory mechanism was further validated in immunodeficient mouse models (Figure 6; Figure S6). The adjuvant effect of the non‐targeting shRNA–encoding DNA vaccine was abolished in MAVS^−^/^−^ and Casp1^−^/^−^ mice but retained in TLR3^−^/^−^ mice, indicating mediation through the IRE1α–RIDD–RIG–I signaling axis. In the AGB6 mouse model, the non‐targeting shRNA–encoding DNA vaccine conferred superior protection against ZIKV infection, as evidenced by reduced viremia, elevated neutralizing antibody titers, and improved survival compared to controls.

Prior research has demonstrated that short interfering RNAs (siRNAs) can be deliberately engineered to act as immunostimulatory adjuvants, enhancing immune responses across vaccine, antiviral, and cancer therapy applications. For example, immunostimulatory or “bifunctional” siRNAs have been shown to enhance dendritic cell (DC) activation and T‐cell responses by simultaneously targeting immunosuppressive genes and activating innate immune sensors [20]. In DC‐based cancer vaccines, an siRNA targeting the enzyme IDO (a negative regulator of T‐cell immunity) not only silenced this immunosuppressive pathway but also directly matured DCs and promoted T‐cell activation [21], correlating with improved clinical outcomes when IDO‐silenced DC vaccines were given to patients [22]. In antiviral settings, incorporating specific sequence motifs into siRNAs has proven effective; a classic example is the GU‐rich 5′‐UGUGU‐3′ motif, which engages endosomal TLR7/8 signaling and dramatically elevates type I interferon and other cytokines, thereby amplifying the siRNA's antiviral efficacy beyond its direct gene‐silencing action [23]. Similarly, structural optimizations such as adding a non‐pairing uridine bulge in the siRNA duplex can broadly enhance cytokine induction (e.g., greater than tenfold increases in TNF‐α and IFN‐α) without compromising target knockdown, conferring greater protection in viral infection models compared to unmodified siRNAs [24].

Notably, most prior approaches rely on defined RNA sequences or chemical modifications to engage pattern recognition receptors such as TLR7/8 or PKR [25], requiring careful optimization to balance gene‐silencing activity with innate stimulation. In contrast, the present study offers a conceptually distinct strategy: using a non‐targeting shRNA that intrinsically activates the IRE1α–RIDD pathway, leading to downstream RIG‐I signaling. This approach obviates the need for any sequence‐encoded immunostimulatory motif or exogenous chemical modification and, importantly, does not require knocking down a host gene to exert its adjuvant effect. By harnessing a specific ER stress sensor (IRE1α) to generate immunostimulatory RNA decay products, our work builds upon the foundation of RNA‐based adjuvants but extends it into a new paradigm of intrinsic adjuvanticity. This complementary mechanism simplifies the vaccine construct while achieving potent innate immune activation, highlighting how leveraging the IRE1–RIDD–RIG–I axis can elicit robust vaccine adjuvant effects without the sequence constraints of earlier methods.

Currently, DNA vaccine development is constrained by delivery challenges and low immunogenicity, often requiring high doses (2–4 mg per dose) for efficacy in humans [2]. Nonetheless, the ZyCoV‐D vaccine—administered intradermally via needle‐free jet injection—was granted emergency use authorization in India for COVID‐19 in 2021 [26]. Electroporation remains a more efficient delivery strategy, although clinical‐use devices remain limited [27]. Recently, an electroacupuncture‐based electroporation device was approved for clinical testing in Taiwan [28]. Our intrinsic adjuvant strategy achieved protective neutralizing antibody titers with only one‐eighth the DNA dose, supporting its utility in dose‐sparing vaccine designs (Figure 5D). These advancements enhance the feasibility of using DNA vaccines in clinical settings.

Overall, this study contributes to the growing body of knowledge on DNA vaccine adjuvant design, offering a novel approach to RNA‐mediated immune modulation using non‐targeting shRNA. This strategy has broad implications for enhancing vaccine efficacy against ZIKV and potentially other flaviviruses, emerging pathogens, or cancers. Future work should focus on evaluating long‐term safety and durability of immune responses, as well as on further optimizing non‐targeting shRNA sequences and dosages to maximize immunogenicity and translational potential.

## 4 Experimental Section/Methods

### 4.1 Cells and Viruses

African green monkey kidney cells (Vero; ATCC, CCL‐81) were used in focus‐forming assays to quantify Zika virus (ZIKV) viral loads in mouse serum. Human embryonic kidney (HEK) 293T cells (ATCC, CRL‐3216) were used for siRNA transfection to assess protein expression of RIDD‐related genes. The RIG‐I reporter cell line, HEK‐Lucia RIG‐I (InvivoGen; Cat. # hkl‐hrigi), was used in luciferase activity assays following siRNA transfection to evaluate RIG‐I pathway activation. The TLR3‐IFN‐β‐Luc reporter cell line, derived from HEK293 cells (provided by Dr. Tsung‐Hsien Chuang, NHRI, Taiwan) [29], was generated by stable transfection with a TLR3 expression vector and an IFN‐β promoter‐driven MetLuc reporter construct (Clontech Laboratories, Inc.). The MetLuc reporter encoded secreted *Metridia* luciferase, and luciferase activity released into the culture medium was measured using the Ready‐To‐Glow Secreted Luciferase Reporter System (Clontech Laboratories, Inc.) according to the manufacturer's instructions. Vero, 293T, HEK‐Lucia RIG‐I, and TLR3‐IFN‐β‐Luc cells were cultured in Dulbecco's Modified Eagle's Medium (DMEM) supplemented with 10% fetal bovine serum (FBS; Gibco BRL, CA, USA) at 37°C in a 5% CO_2_ incubator. The mouse monocyte cell line JAWSII (ATCC, CRL‐11904) was used for cytokine analysis under siRNA transfection and was cultured in α‐minimum essential medium (α‐MEM) supplemented with 20% FBS and 5 ng/ml murine GM‐CSF at 37°C in a 5% CO_2_ incubator. All cell lines were regularly tested and confirmed to be free of contamination during experimental procedures.

The ZIKV epidemic 2015 Puerto Rican strain (PRVABC59; GenBank accession KU501215) was obtained from the Centers for Disease Control (CDC) of Taiwan. Virus propagation was performed in Vero cells, and viral titers were determined by focus‐forming assays (FFU/mL).

### 4.2 Mice

All animals were housed at the Animal Center of the National Health Research Institutes (NHRI) and maintained in accordance with institutional animal care protocols. C57BL/6 mice were procured from the National Laboratory Animal Breeding and Research Center in Tainan, Taiwan. TLR3‐deficient (TLR3^−/−^) mice [30, 31], B6N.129S2‐Casp1/J /J (Casp1‐/‐) mice, and B6;129‐Mavstm1Zjc/J /J (MVAS^−/−^) mice were obtained from the Jackson Laboratory (Bar Harbor, ME, USA). The AGB6 (C57BL/6 background) immunocompromised mice with Type I and II interferons (IFN‐α/β and γ) receptor‐deficient were obtained from the Jackson Laboratory (Bar Harbor, ME, USA) [32]. All mice were bred at the Laboratory Animal Center of the NHRI. Mice aged 6 and 8 weeks were selected for the experiments. Viral challenge studies were conducted in BSL‐2 animal facilities in accordance with applicable guidelines. The Institutional Animal Care and Use Committee (IACUC) of the NHRI approved all experimental animal protocols.

### 4.3 Cytokine Analysis

The non‐targeting siRNAs (NTsiRNA‐1: UUCUCCGAACGUGUCACGUTT, NTsiRNA‐2: CCUAAGGUUAAGUCGCCCUCG) were obtained from MDBio, Inc., and RNA Technology Platform and Gene Manipulation Core, Taiwan, respectively, and were delivered into JAWSII mouse dendritic cells (DCs) using the transfection reagent TransIT‐X2 (Mirus Bio). Forty‐eight hours post‐transfection, cell‐free supernatants were harvested and stored at −80°C. The TNFα, IL‐6, and IL‐1β levels were measured using the TNFα ELISA Kit (Invitrogen, cat# 88 7324 88), IL‐6 ELISA Kit (Invitrogen, cat# 88 7064 88), and IL‐1β ELISA Kit (Invitrogen, cat# 88‐7013‐88) according to the manufacturer's instructions.

### 4.4 BMDC Activation

Mouse bone marrow dendritic cells (mBM‐DCs) derived from C57BL/6 mice were cultured at a density of 2 × 10^6^ cells in Petri dishes containing 10 mL of complete RPMI‐1640 supplemented with 200 units/mL (20 ng/mL) recombinant mouse GM‐CSF (PeproTech, Rocky Hill, NJ, USA). An additional 10 mL of complete RPMI containing 20 ng/mL GM‐CSF was added on day 3. The cells were collected from each dish and counted on day 6. mBM‐DCs (1 × 10^6 ^cells/mL) were transfected with 300 pmol of synthetic 5’‐Cy5‐labelled non‐targeting siRNA (TRC or TRC19) by using the transfection reagent TransIT‐X2 (Mirus Bio). After 48 h, cells were collected and stained in FACS buffer with a panel of surface antibodies containing APC‐Cy7/anti‐CD11c, Alexa Fluor 700/anti‐MHC II, FITC/anti‐CD80, and PE/anti‐CD83 for 30 min at 4°C. Cells were washed and fixed with IC Fixation buffer (ThermoFischer Scientific, Waltham, MA, USA) for 20 min at 4°C. Cells were suspended in FACS buffer and detected by acquisition on an Attune NxT Flow cytometer (Invitrogen). All results were analyzed using FlowJo v.10.0.

### 4.5 Luciferase Activity Assay

HEK‐Lucia RIG‐I or TLR3‐IFNβ‐Luc HEK293 cells were seeded at 5 × 10^5^ cells/well in a 6‐well plate containing 2 mL of complete culture medium and incubated overnight at 37°C with 5% CO_2_. The following day, cells were transfected with 5’‐Cy5‐labelled non‐targeting siRNAs using TransIT‐X2 reagent (Mirus Bio) according to the manufacturer's instructions. After 12–48 h of transfection, 50 µL of culture supernatant was collected from each well and transferred to a 96‐well plate. An equal volume (20 µL) of QUANTI‐Luc 4 Lucia Reagent was added to each well for luminescence measurement. The secreted luciferase activity was quantified using a microplate reader.

### 4.6 Plasmid DNA Constructs

The ZIKV prME sequence obtained from the PRVABC59 strain (isolated in March 2015 in Puerto Rican) was codon‐optimized for mammalian expression, and the signal sequence was replaced with that of JEV. The designed ZIKV prME gene containing a Kozak sequence was synthesized by Genscript (Piscataway, NJ, USA) and subcloned through *NheI* and *NotI* into the pVax1 vector (Thermo‐Fisher, Waltham, MA, USA) to generate pVax‐prME. The U6 promoter sequence and the scramble shRNAs were inserted into pVax‐prME via MluI and NruI to generate pVax‐TRC‐prME and pVax‐TRC19‐prME. All plasmid DNA was amplified by *E. coli* DH5α competent cells (ThermoFisher, Waltham, MA, USA) and purified using an EndoFree plasmid purification kit (Qiagen, Redwood, CA, USA).

### 4.7 Antigen Expression and Protein Detection

A monolayer of HEK293T cells was cultured on a 6‐well plate and incubated in 5% CO_2_ at 37°C. The non‐targeting siRNAs (NTsiRNA‐1 and NTsiRNA‐2) were delivered by the transfection reagent TransIT‐X2 (Mirus Bio). Six hours post‐transfection, HEK293T cells were harvested, and cell lysates were resolved by running SDS‐PAGE. The separated proteins were transferred to a PVDF membrane and incubated with mouse antibodies: IRE1α (#3294, Cell Signaling), IRE1‐pS724 (ab48187, Abcam), NPGPx (85120‐1‐RR, Proteintech), TXNIP (ab188865, Abcam), GRP78 (ab21685, Abcam), and GAPDH (81640‐5‐RR, Proteintech). The purified plasmid DNA was delivered into cells using the transfection reagent PolyJet (Cat. # SL100688, SignaGen). After 3 days, HEK293T cells were harvested, and cell lysates were resolved by running SDS‐PAGE. The separated proteins were transferred onto a PVDF membrane and incubated with mouse anti‐ZIKV Env monoclonal IgG (Cat. # MABF2046, MILLIPORE, CA, USA). The expression of GAPDH as an internal control was detected using a rabbit anti‐GAPDH IgG (Cat. # 10494‐1‐AP, Proteintech, IL, USA). HRP‐conjugated polyclonal goat anti‐mouse antibody was used as the secondary antibody (Cat. # 31430, Thermo Fisher, MA, USA).

### 4.8 Animal Immunizations

The mice were shaved in the area of the gastrocnemius muscle, which was then injected with the indicated plasmid DNA in 100 µL of water before electroporation (EP) by an electroporator (ECM830) using two‐needle array electrodes with 5‐mm diameter (BTX, Holliston, MA, USA). Intramuscular electroporation was performed at a constant voltage of 75 V with 10 pulses at 50 msec/pulse and 100 msec intervals between pulses, as previously described [33]. After immunization with two doses at 3‐week intervals, mouse blood samples were collected via submandibular venous sampling.

### 4.9 Enzyme‐Linked Immunosorbent Assay (ELISA)

ELISA was used to measure IgG titers against the ZIKV envelope in the serum of vaccinated mice. Each well on 96‐well microplates (Corning, Glendale, Arizona, USA) was coated with 0.4 µg of purified ZIKV Env protein in Phosphate‐buffered saline (PBS) and incubated at 4°C overnight. After a blocking step, serum was diluted 1:20 and then serially diluted twofold in 1% FBS in PBS, and the mixture was plated in wells for 3 h of incubation at room temperature. The wells were washed three times with PBS containing 0.05% Tween 20 (PBS‐T), then incubated with goat anti‐mouse IgG‐HRP (Cappel, Solon, OH, USA) at a 1:5000 dilution with 1% FBS in PBS for 1 h at room temperature. The signals were developed by loading TMB substrate (Seracare, Milford, MA, USA) into each well, and the reaction was stopped by adding 1N H_2_SO_4_. The absorbance at 450 nm was obtained by a microplate reader (BioTek, Winooski, VT, USA). The cutoff (endpoint titer) was determined from the negative‐control serum titer and its standard deviation. The reciprocal of the penultimate serum dilution above the cut‐off was taken as the antibody titer.

### 4.10 Enzyme‐Linked ImmunoSpot (ELISPOT) Assay

The levels of interferon‐gamma (IFN‐γ) produced by the splenocytes of immunized mice were detected by a mouse IFN‐γ ELISPOT assay kit (BD Biosciences, San Jose, CA, USA) according to the manufacturer's instructions. Briefly, a 96‐well plate with PVDF membranes (Merk & Millipore, Burlington, MA, USA) was incubated with the capture antibody (1/200) at 4°C overnight, then blocked with complete RPMI‐1640 medium at 37°C for 2 h. Splenocytes (5 × 10^5^/well) were seeded and stimulated by incubation with 10 µg/mL recombinant Env or with synthetic peptides representing CD4 and CD8 epitopes in C57BL/6 mice [34, 35]. After 48 h, the plate was washed twice with PBS‐T and incubated with a biotinylated detection antibody (1/250) for 2 h. After washing with PBS‐T, streptavidin‐HRP (1/100) was incubated for 1 h. Spots were developed using an AEC substrate reagent (BD Bioscience, NJ, USA) and counted by an ELISPOT reader (Cellular Technology Ltd., Shaker Heights, OH, USA).

### 4.11 Focus‐Forming Assays

Vero cells (1 × 10^5^ cells/well) were cultured in 24‐well plates at 37°C for 24 h. Beginning at 1:10, 10‐fold serial dilutions of plasma were mixed with equal volumes of ZIKV (PRVABC59) for 4 h at 4°C. Subsequently, duplicate dilutions were applied to cells in each well for 3 h at 37°C. Following aspiration of the virus/plasma mixture, the cells were washed with PBS and covered with 8% methylcellulose in DMEM medium supplemented with antibiotics and 2% fetal calf serum (FBS). After a two‐day incubation at 37°C, the methylcellulose overlay was removed by aspiration, followed by rinsing with PBS. After being fixed for more than 15 min with 4% formaldehyde, the cell monolayer was permeabilized with 0.1% nonidet P40/PBS for 15 min, and blocked with 3% bovine serum albumin/PBS for 15 min. ZV‐infected Vero cells were identified using the HB122 ZIKV‐specific antibody (1/5000) and HRP‐conjugated goat anti‐mouse IgG (1/7000, Thermo 34 310). A TMB precipitating reagent (ScyTek, UT, USA) was added to develop pigmentation in infected cells, which can be visualized and quantified to determine viral titers with focus‐forming units (FFUs).

### 4.12 Focus Reduction Neutralization Tests (FRNT)

To achieve 50% confluence and facilitate focus‐reduction neutralization titer (FRNT) 50 assays, Vero cells (1 × 10^5^ cells/well) were cultured in 24‐well plates at 37°C for 24 h. Beginning at 1:8, twofold serial dilutions of heat‐inactivated serum were mixed with equal volumes of ZIKV (PRVABC59) in a final volume of 200 µL at 4°C overnight. Subsequently, duplicate dilutions were applied to cells in each well for 3 h at 37°C. Follow the focus‐forming assay described before to determine the FFUs of each well. The percentage reduction in FFUs was estimated by comparing samples with the same dilutions of control serum from unimmunized animals. The highest dilution that resulted in a 50% reduction in FFUs was defined as the FRNT_50_ neutralizing antibody titer. Any neutralizing antibody titers below 8 were designated as 4 for calculation purposes.

### 4.13 ZIKV Challenge in AGB6 Mice

In the Zika virus challenge studies, AGB6 mice were intramuscularly vaccinated at weeks 0 and 3 with the indicated dose of plasmid DNA, and the control group received an equivalent dose of the pVAX1 vector. Before the viral challenge, serum samples were collected from all mice at week 5 to assess anti‐Env IgG titers and neutralizing antibody titers. AGB6 mice were challenged with 50 FFU of ZIKV (PRVABC59) in 0.2 mL of PBS via the intraperitoneal (IP) route on week 6. On day 3 post‐infection, plasma samples from all mice in each group were collected for viremia assessment using the FRNT50 assay. All groups were observed daily for clinical signs of disease. The criteria for euthanasia on welfare grounds consisted of 20% weight loss or prolonged paralysis in one or both hind limbs.

### 4.14 Viremia of ZIKV

Blood samples from all AGB6 mice challenged in each group were collected at 3 dpi. The blood (0.2 mL) was immediately mixed with 0.02 mL of 3.8% sodium citrate pre‐chilled on ice. The plasma was isolated by centrifugation at 5000 rpm for 5 min, and viremia was assessed by focus‐forming assays in Vero cells. Any infective titers below the detection limit (2 log10 FFU/mL) were assigned a value of 1 for calculation purposes.

### 4.15 Data Analysis

All statistical analyses were performed using GraphPad Prism. A two‐tailed Mann–Whitney test was used to compare experimental data between the two vaccine groups. Besides, the Kruskal–Wallis analysis of variance (ANOVA) was also used as a secondary test, performing multiple comparisons across all groups. A two‐way ANOVA was applied to multiple groups across different time points. *P‐*values < 0.05 were considered statistically significant. The significance of the survival rates was assessed by the survival curve test.

### 4.16 Bioinformatics and Gene Set Enrichment Analysis (GSEA)

To mechanistically interpret the transcriptomic signatures associated with non‐targeting siRNA transfection and to infer the functional engagement of the RNA‐induced silencing complex (RISC), GSEA was performed. Differential expression data from JAWSII cells transfected with NTsiRNA‐1 or NTsiRNA‐2, compared with the Mock control, were used to generate ranked gene lists in descending order of log2 fold change (log2FC). GSEA was conducted utilizing the clusterProfiler R package [36]. Reference gene sets for Mus musculus were retrieved from the Molecular Signatures Database (MSigDB) [37] using the msigdbr package [38].

To evaluate specific hypotheses regarding the mechanism of action, the following gene sets were analyzed: (i) “RNA silencing machinery and processing” (GO:0031047) to assess the transcriptional compensation of RISC components; (ii) “miR‐17 Targets” (MSigDB C3: MIRDB) to validate the functional consequences of IRE1α‐mediated RIDD activity; (iii) “Hallmark Unfolded Protein Response” (MSigDB H) and “response to endoplasmic reticulum stress” (GO:0034976) to distinguish specific RIDD activation from generalized ER stress; and (iv) “KEGG RNA Degradation” (MSigDB C2) and “Reactome Nonsense Mediated Decay” (MSigDB C2) to evaluate global RNA decay activity. The normalized enrichment score (NES) and Benjamini–Hochberg‐adjusted *p*‐values were calculated to assess statistical significance and the direction of pathway regulation. Visualization of enrichment plots, leading‐edge subsets, and log2FC rankings was performed using the enrichplot and ggplot2 packages.

## Conflicts of Interest

The authors declare no conflicts of interest.

## Supporting information




**Supporting File**: advs74034‐sup‐0001‐SuppMat.docx.

## Data Availability

The data that support the findings of this study are available from the corresponding author upon reasonable request.
