# Supplementary material for: Non‐Targeting shRNA‐Encoded Plasmid DNA Enhances Protective Immunity Through RIDD‐RIG‐I Signaling Pathway in the Zika Virus Animal Model
Source: Adv Sci (Weinh). 2026 Jan 28;13(19):e17420. doi: 10.1002/advs.202517420 (PMC13045247; doi:10.1002/advs.202517420)
Supplement: Supplementary file 1 — Supporting File: advs74034‐sup‐0001‐SuppMat.docx. [file ADVS-13-e17420-s001.docx]

**Supporting Information**

**Non-targeting shRNA encoded plasmid DNA enhances protective immunity through RIDD-RIG-I signaling pathway in the Zika virus animal model**

*Min-Syuan Huang, Hung-Chun Liao, Po Peng, Wan-Ling Wu, Kit Man Chai, Mei-Yu Chen, Guann-Yi Yu, Tsung-Hsien Chuang, Hsin-Wei Chen, Chuang-Rung Chang, and Shih-Jen Liu**


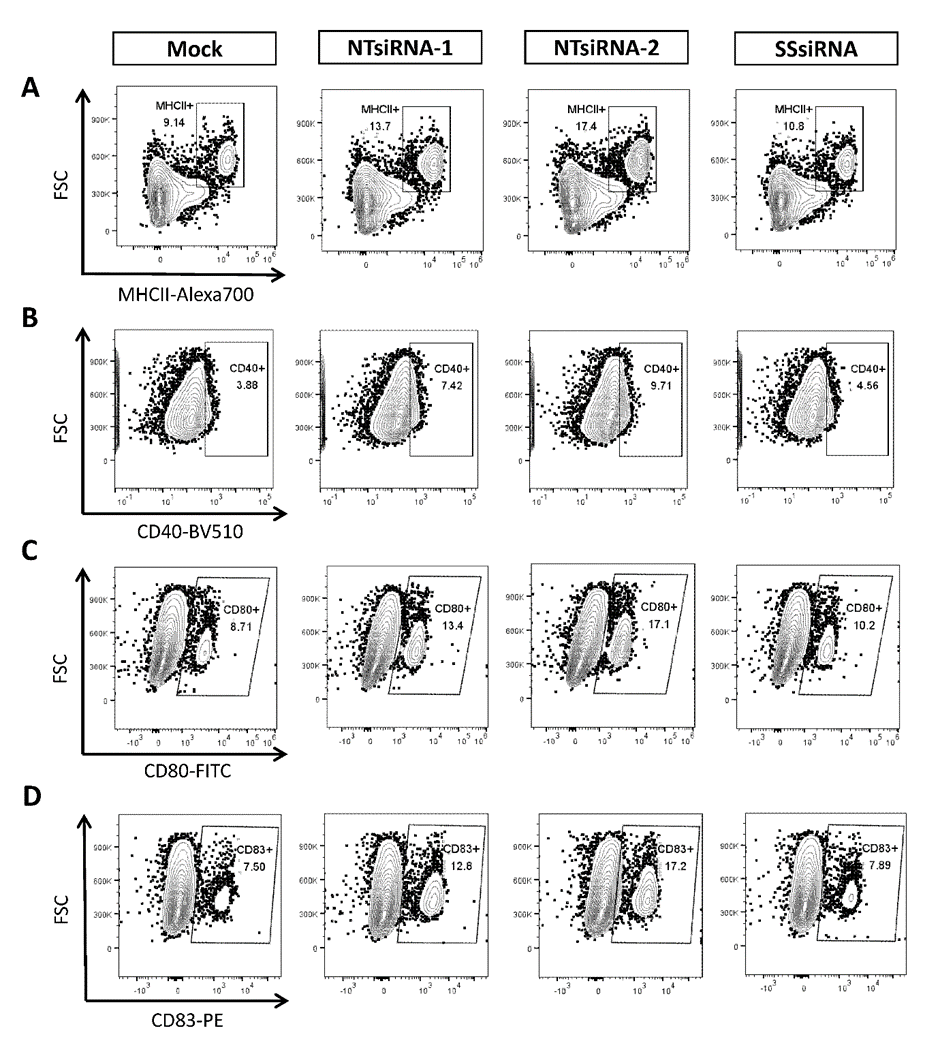


**Figure S1. Non-targeting siRNA transfection upregulated co-stimulatory molecules on mBM-DCs.**

Representative gating of (**A**) MHC II, (**B**) CD40, (**C**) CD80, and (**D**) CD83 expression from BM-DCs CD11c+ cells after coculturing with transfection reagent (Mock control) or transfected with NTsiRNA-1, NTsiRNA-2, or SSsiRNA for 48 hours.


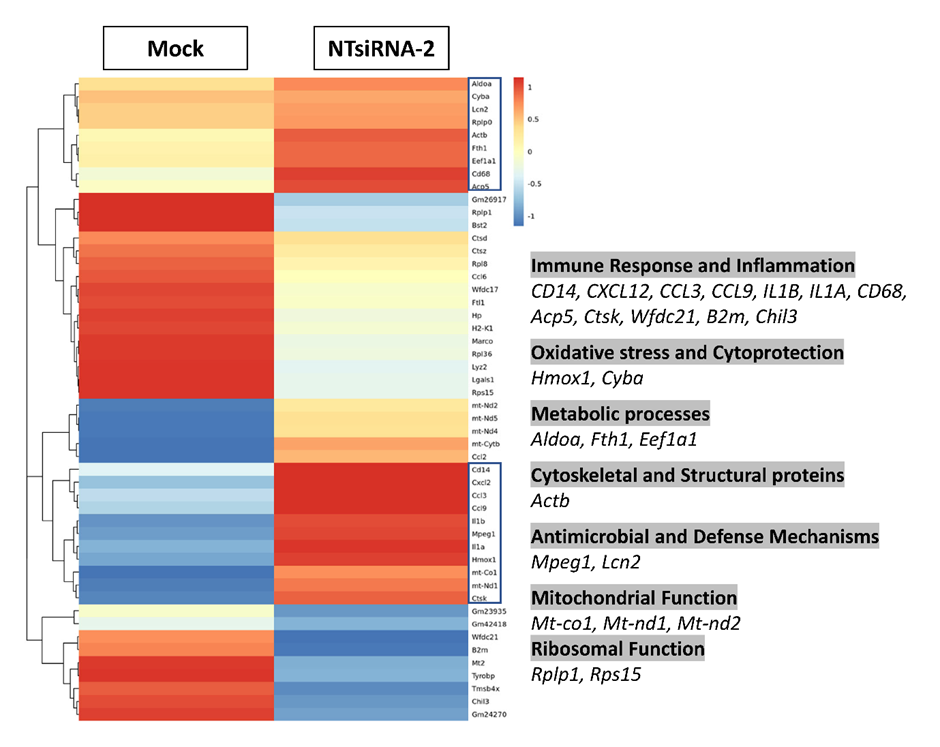


**Figure S2. Heatmap of the top 50 DEGs between JAWSII cells transfected with or without non-targeting siRNA.**

To visualize the global transcriptomic alterations induced by non-targeting siRNA (TRC) treatment, a heat map of the top 50 differentially expressed genes (DEGs) was generated using RNA-Seq analysis. In this heatmap, the horizontal axis represents samples, and the vertical axis represents genes. The color scale (red to blue) represents gene expression levels between the mock control and NTsiRNA-2 transfection, with red indicating increased expression and blue indicating decreased expression.


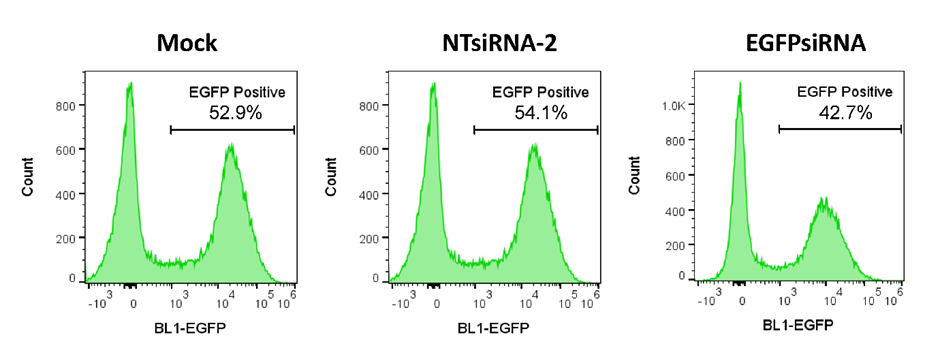


**Figure S3. EGFPsiRNA transfection reduced the ratio of EGFP-expressing HEK-Lucia RIG-I/EGFP cells.**

HEK-Lucia RIG-I/EGFP cells were transfected with NTsiRNA2 or EGFPsiRNA (EGFP-targeting siRNA). Flow cytometric analysis at 48 hours post-transfection revealed a significant reduction in EGFP-positive cell population, indicating detectable EGFP silencing.


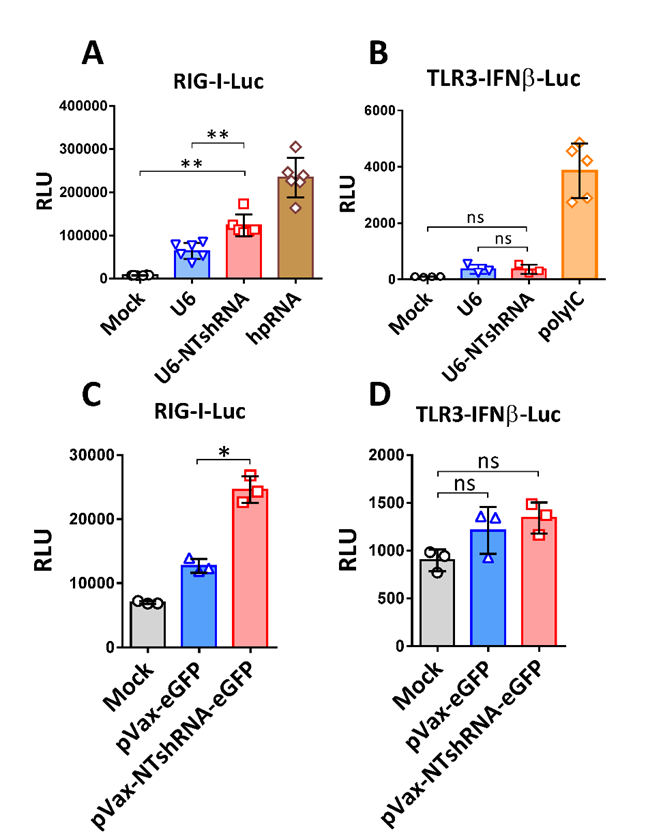


**Figure S4. DNA-derived non-targeting shRNA expression activated RIG-I but not TLR3 signaling.**

(A) HEK-Lucia RIG-I cells and (B) TLR3-IFNβ-Luc expressed HEK293 cells were transfected with U6, U6-NTshRNA PCR products, and positive controls (hpRNA or polyIC) for 48 hours, then supernatants were collected to detect the luciferase activity. (C) HEK-Lucia RIG-I cells and (D) TLR3-IFNβ-Luc expressed HEK293 cells were transfected with eGFP and NTshRNA-eGFP plasmids for 24 hours. The eGFP-expressing cells were then sorted to detect luciferase activity, with 1 × 10^6 cells per group after 24 hours of culture. Data are presented as mean ± SD from three independent experiments. Significance was calculated by Mann-Whitney two-tailed t-test comparisons. * p < 0.05, ** p < 0.005, and ns = no significant.


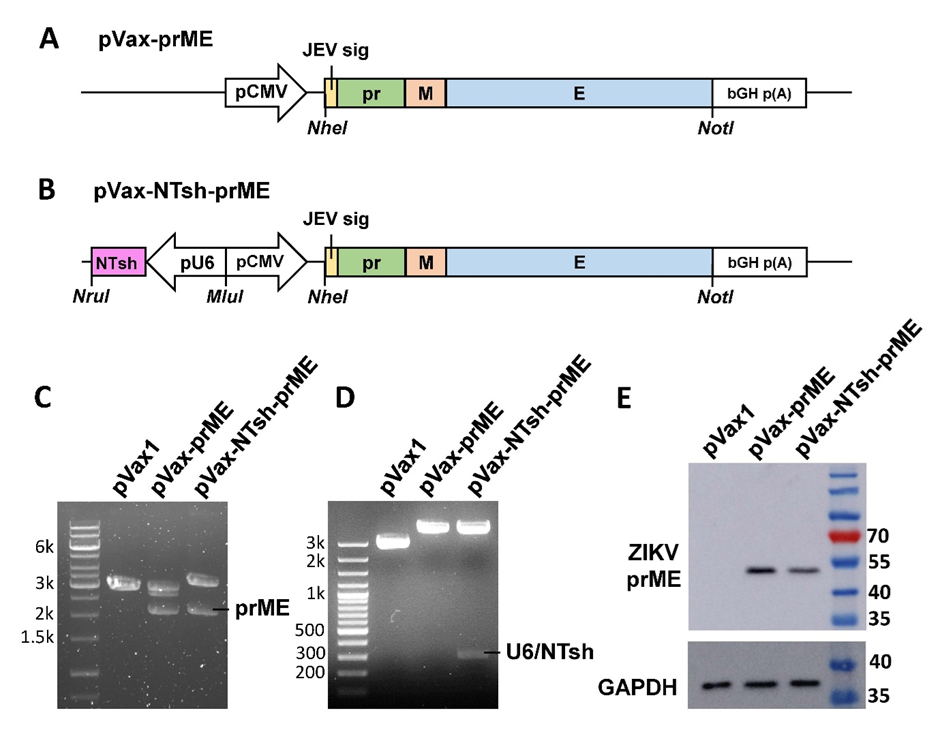


**Figure S5. Construction and characterization of DNA vaccine encoding non-targeting shRNA and ZIKV prME.**

(A) Schematic diagram of pVax-prME that encodes a JEV signal peptide followed by recombinant ZIKV prME structural protein. (B) pVax-NTsh-prME was designed by inserting a U6 promoter and the non-targeting siRNA-2’s sequence of shRNA upstream of pCMV in pVax-prME. Agarose gel electrophoresis of the plasmid DNA of pVax1, pVax-prME, and pVax-NTsh-prME after double digestion by restriction enzymes (C) NheI and NotI (D) NruI and MluI. (E) Western blot analysis was performed to detect prME expression using a mouse anti-ZIKV ENV antibody, and GAPDH was used as an internal control with a goat anti-GAPDH antibody.


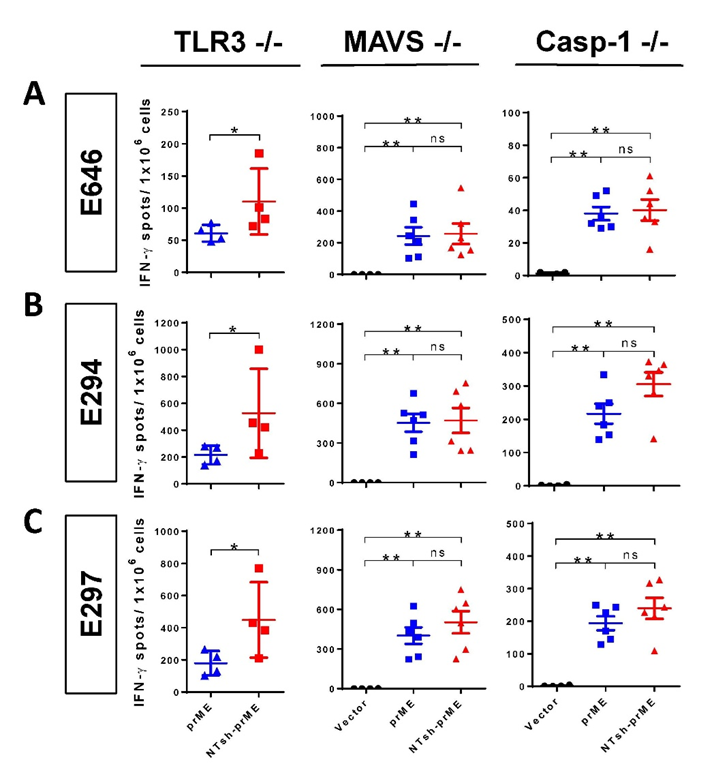


**Figure S6. Non-targeting shRNA enhances the T-cell-mediated immune responses of ZIKV DNA vaccine in TLR3 KO mice but not in MAVS KO and Caspase-1 KO mice.**

TLR3 KO, MAVS KO, and Caspase-1 KO mice (N= 4~6 per group) were i.m. immunized with 5μg DNA of pVax-prME, or pVax-NTsh-prME by electroporation at weeks 0 and 3. Splenocytes were isolated to analyze antigen-specific T-cell immune responses. After the stimulation of ZIKV Env epitopes (**A**) E646, (**B**) E294, and (**C**) E297 for 48 hours, the frequency of epitope-specific IFN-γ-secreting T cells (spot-forming units per million splenocytes) induced in vaccinated mice was determined by IFN-γ ELISpot assay. Each point represents individual animals, each bar reflects the mean of titers, and error bars represent the standard deviation. Significance was calculated by Mann-Whitney one-tailed t-test comparisons. * p < 0.05, **P ≤ 0.01, and ns = no significant.


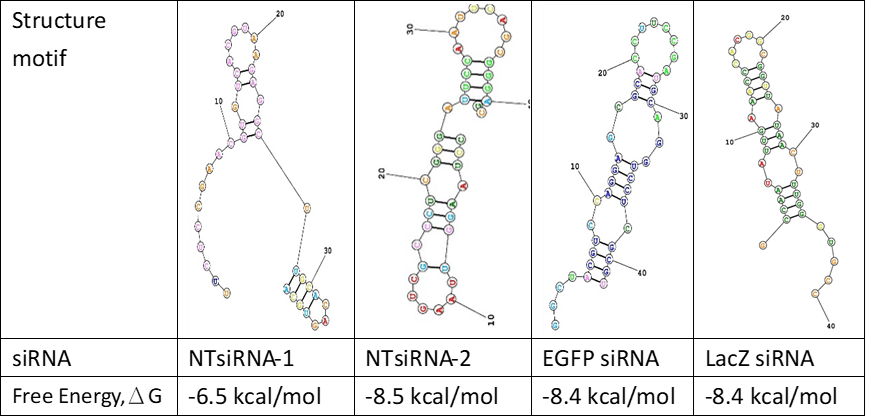


**Figure S7. Sequence motif arrangement and predicted structural features of siRNAs used in this study.**

Schematic illustration of sequence motif distribution and predicted secondary structures of NTsiRNA-1, NTsiRNA-2, EGFP siRNA, and LacZ siRNA. GU-containing di-nucleotides and short GU-rich motifs are annotated along each siRNA strand to indicate their relative positions within the duplex. RNA secondary structures were predicted using the RNAstructure web-based software (RNAstructure, University of Rochester), a publicly available RNA folding platform, and the corresponding minimum free energy (MFE; ΔG, kcal/mol) values are shown for each siRNA. All four siRNAs exhibited comparable predicted structural stability, with MFE values ranging from −6.5 to −8.5 kcal/mol. No enrichment or preferential localization of known immunostimulatory motifs within specific structural elements (e.g., stem or loop regions) was observed. These analyses indicate that differences in motif arrangement or in predicted secondary-structure stability are unlikely to account for the observed innate immune activation, supporting a sequence-independent mechanism.


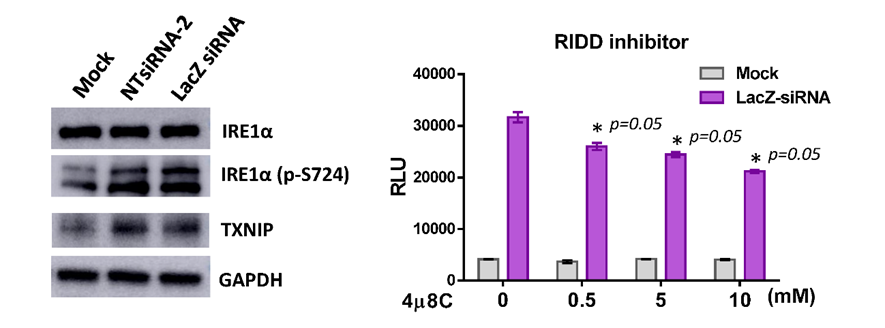


**Figure S8. Sequence-distinct non-targeting LacZ siRNA activates the IRE1α–RIDD–RIG-I signaling axis.**

(A)HEK293 cells were transfected with NTsiRNA-2 and LacZ siRNA for 24 hours, and the cell lysates were collected for western blotting analysis. The expression levels of IRE1α, IRE1α (p-S724), TXNIP, and GAPDH were detected by using specific antibodies. (B)HEK-Lucia RIG-I cells were pretreated with 0.5 mM, 5 mM, and 10 mM KIDD inhibitor (4μ8C) for 6 hours, then transfected with LacZ siRNA for 24 hours; supernatants were collected to measure luciferase activity. Data are presented as mean ± SD from three independent experiments. Significance was calculated by Mann-Whitney two-tailed t-test comparisons. * p < 0.05, ** p < 0.005, and ns = no significant.
